# Chromosome-level phased genome assembly of “Antonovka” identified candidate apple scab-resistance genes highly homologous to *HcrVf2* and *HcrVf1* on linkage group 1

**DOI:** 10.1093/g3journal/jkad253

**Published:** 2023-11-04

**Authors:** Anže Švara, Honghe Sun, Zhangjun Fei, Awais Khan

**Affiliations:** Plant Pathology and Plant-Microbe Biology Section, School of Integrative Plant Science, Cornell University, Geneva, NY 14456, USA; Boyce Thompson Institute, Cornell University, Ithaca, NY 14853, USA; Plant Biology Section, School of Integrative Plant Science, Cornell University, Ithaca, NY 14853, USA; Boyce Thompson Institute, Cornell University, Ithaca, NY 14853, USA; USDA-ARS Robert W. Holley Center for Agriculture and Health, Ithaca, NY 14853, USA; Plant Pathology and Plant-Microbe Biology Section, School of Integrative Plant Science, Cornell University, Geneva, NY 14456, USA

**Keywords:** *Malus domestica*, *Venturia inaequalis*, phased genome assembly, phylogeny, sequence alignment, haplotypes, Plant Genetics and Genomics

## Abstract

Apple scab, a fungal disease caused by *Venturia inaequalis*, leads to losses in both yield and fruit quality of apples (*Malus domestica* Borkh.). Most commercial apple cultivars, including those containing the well-characterized *Rvi6-*scab-resistance locus on linkage group (LG) 1, are susceptible to scab. *HcrVf2* and *HcrVf1* are considered the main paralogs of the *Rvi6* locus. The major apple scab-resistance loci *Vhc1* in “Honeycrisp” and *Rvi17* in “Antonovka,” were identified in close proximity to *HcrVf2*. In this study, we used long-read sequencing and in silico gene sequence characterization to identify candidate resistance genes homologous to *HcrVf2* and *HcrVf1* in Honeycrisp and Antonovka. Previously published chromosome-scale phased assembly of Honeycrisp and a newly assembled phased genome of Antonovka 172670-B were used to identify *HcrVf2* and *HcrVf1* homologs spanning *Vhc1* and *Rvi17* loci. In combination with 8 available *Malus* assemblies, 43 and 46 DNA sequences highly homologous to *HcrVf2* and *HcrVf1*, respectively, were identified on LG 1 and 6, with identity and coverage ranging between 87–95 and 81–95%, respectively. Among these homologs, 2 candidate genes in Antonovka and Honeycrisp haplome A are located in close physical proximity to the scab-resistance marker Ch-Vf1 on LG 1. They showed the highest identity and coverage (95%) of *HcrVf2* and only minor changes in the protein motifs. They were identical by state between each other, but not with *HcrVf2*. This study offers novel genomic resources and insights into the *Vhc1* and *Rvi17* loci on LG 1 and identifies candidate genes for further resistance characterization.

## Introduction

Apples (*Malus domestica* Borkh.) are the most-produced fruit crop grown in temperate climates globally. Over the last 3 decades, the annual global production of apples has more than doubled, from 41 to 93 million tons (FAOSTAT 2021). Extensive pesticide application, improved horticultural practices, and breeding are among the major drivers of increased apple production (Khan and Korban 2022). Although pesticides are highly efficient in ensuring the stable production of disease-free apples, their overuse over the last few decades has had adverse effects on biodiversity and human health (Zaller and Brühl 2019; Hernández *et al.* 2020). In the last few decades, apple breeding programs have achieved major improvements in consumer-preferred fruit quality, horticultural traits, and storability; however, advances in disease resistance have been limited (Khan and Korban 2022).

Apple scab caused by *Venturia inaequalis* is the most economically significant fungal disease of apples and is highly challenging to control. The life cycle of *V. inaequalis* is complex, as it is divided into sexual ascospore production in spring followed by asexual conidia production in spring and summer, both resulting in obvious black, gray, or brown infection lesions on susceptible leaves and fruits (MacHardy 1996). During the growing season, 15–20 fungicide spray applications and/or the implementation of scab-resistant cultivars with a single qualitative resistance gene, such as *Rvi6* from *Malus floribunda* 821, are required for successful scab control (MacHardy 1996; Turechek 2004; Bus *et al.* 2011). Frequent fungicide application and deployment of monogenic resistance to control scab have resulted in the selection of pesticide-tolerant and *Rvi*-overcoming fungal strains in all major apple production areas (Parisi *et al.* 1993; Shirane *et al.* 1996; Köller and Wilcox 2000; Köller *et al.* 2002, 1997; Turechek and Köller 2004; Bus *et al.* 2011; Beckerman *et al.* 2013; Papp *et al.* 2019; Patocchi *et al.* 2020). To ensure sustainable scab control, identification and characterization of additional scab-resistance genes have been proposed as a need to develop cultivars with more durable scab resistance (Bus *et al.* 2011; Khajuria *et al.* 2018).

To date, 19 major-effect apple scab-resistance loci have been identified and termed as *Rvi* genes (Bus *et al.* 2011; Patocchi *et al.* 2020). These loci provide resistance against the majority of the known *V. inaequalis* strains. A few of these genes, including *Rvi5*, *-11*, *-12*, *14*, and *-15*, still result in scab resistance in the host in the majority of apple-producing regions (Patocchi *et al.* 2020). Furthermore, candidate resistance genes of *Rvi1*, *Rvi6*, *Rvi12*, and *Rvi15* have been identified, among which *Rvi6* and *Rvi15* have been functionally characterized (Belfanti *et al.* 2004; Malnoy *et al.* 2008; Joshi *et al.* 2011; Schouten *et al.* 2014; Cova *et al.* 2015; Padmarasu *et al.* 2018). These studies have shown that the genetic structure of the characterized loci is complex and harbors multiple gene paralogs and/or genes without known resistance functions (Vinatzer *et al.* 2001; Galli *et al.* 2010; Bus *et al.* 2011; Bastiaanse *et al.* 2016).

The distal end of linkage group (LG) 1 of apples harbors *Rvi6* from *M. floribunda* 821 and 2 tightly co-located qualitative resistant genes, *Rvi17* present in “Antonovka” and *Vhc1* from “Honeycrisp” apples. *Rvi6* is the most widely used and well-characterized apple scab-resistance locus. However, the relationship between *Rvi17* and *Vhc1* remains unclear. *Rvi6* harbors 4 paralogs, *HcrVf1*-*4*, of which *HcrVf2* unanimously confers resistance to a wide range of *V. inaequalis* strains (Belfanti *et al.* 2004; Malnoy *et al.* 2008; Joshi *et al.* 2011). The resistance allele can be identified using a 159-bp microsatellite marker (CH-Vf1) allele for the selection of progeny carrying *Rvi6*-scab resistance (Tartarini *et al.* 1999; Vinatzer *et al.* 2004). This resistant allele encodes a constitutively expressed leucin-rich repeat receptor-like protein (LRR-RLP), homologous to *HcrVf1*, *-3*, and *-4*, whose expression is increased 24 h after scab inoculation (Vinatzer *et al.* 2001; Malnoy *et al.* 2008). However, the latter alleles have unique polymorphic nucleotides, a number of short random duplications or deletions, and various deletions of complete LRR copy units that hamper their resistance effects (Belfanti *et al.* 2004; Xu and Korban 2004, 2002; Silfverberg-Dilworth *et al.* 2005; Malnoy *et al.* 2008; Broggini *et al.* 2009).

In contrast to *Rvi6*, the underlying alleles and resistance mechanisms of *Rvi17* and *Vhc1* remain unresolved. *Rvi17* is a chlorosis-conditioning locus that is considered instrumental in the complex genetic resistance of “Antonovka Obyknovennaja,” known as the “Common Antonovka” or “Schmidt's Antonovka,” and the related Antonovka accessions (Bus *et al.* 2012; Pikunova *et al.* 2014). It has been suggested to confer resistance against race 6 (Parisi and Lespinasse 1996). The functional allele of *Rvi17* has been associated with the 139-bp amplicon size of CH-Vf1 (Bus *et al.* 2012; Pikunova *et al.* 2014), although this marker occasionally results in inconsistent diagnosis of the *Rvi17*-scab resistance in Antonovka accessions. For example, an apple genotype TN10-8 and a cultivar “Freedom” did not show resistance against *V. inaequalis* race 6 but were able to amplify the 139-bp amplicon of CH-Vf1 (Calenge *et al.* 2004; Bus *et al.* 2012). Similar to *Rvi6*, the defense responses of the *Vhc1* apple scab-resistance locus of Honeycrisp range from no symptoms to sporulation to a broad range in the US *V. inaequalis* strains. The amplicon size of 139 bp of the CH-Vf1 marker (same as in Antonovka) is considered to be diagnostic for the functional allele of the *Vhc1* apple scab-resistance locus (Clark *et al.* 2014).

Genome sequence data can complement genetic mapping for the identification of candidate resistance genes underlying resistance loci. Currently, long-read single-molecule sequencing techniques, such as PacBio long-read sequencing platforms, offer high-quality and cost-effective de novo genome assembly possibilities (Khan and Korban 2022). The long length and high accuracy of sequencing reads have enabled the improved phasing of highly heterozygous genomes (Khan *et al.* 2022). This approach has resulted in the generation of highly contiguous phased genomes of various rosaceous species, including apples (Aranzana *et al.* 2019; Sun *et al.* 2020; Khan *et al.* 2022; Khan and Korban 2022). Phased genome assemblies have enabled the identification and fine-mapping of candidate alleles for disease resistance and/or several other traits in apples and grapevines (Sun *et al.* 2020; Frommer *et al.* 2023). Particularly, the recently published fully phased Honeycrisp assembly (Khan *et al.* 2022) could aid in the identification of apple scab-resistance alleles on LG 1 and the development of molecular markers compared with the existing candidate LG 1 resistance gene identification approaches conducted across different apple accessions (Boudichevskaia *et al.* 2009; Broggini *et al.* 2009). Amplification of *HcrVf*-associated markers in bacterial artificial chromosome genomic libraries derived from “Florina” (Broggini *et al.* 2009) resulted in the identification of 22 homologous sequences on LG 1 and 6, showing 73–94% nucleotide identity with *HcrVf2* (Broggini *et al.* 2009). In addition, 685–748 bp PCR products with 85–100% amino acid sequence identity with *HcrVf1* and *HcrVf2* were identified in cultivars harboring *Rvi6*, other *Rvi* genes and no *Rvi* genes (Boudichevskaia *et al.* 2009). So far, the role of these homologs in scab resistance has not yet been demonstrated.

Here, we developed a phased chromosome-level genome assembly of Antonovka 172670-B (referred to as Antonovka hereafter), and identified homologs of *HcrVf2* and *HcrVf1* apple scab-resistant genes on the distal end of LG 1. We compared *HcrVf* homologs identified in Honeycrisp (Khan *et al.* 2022) and Antonovka genome assemblies with other available apple genomes to identify resistance gene candidates of the *Vhc1* locus in Honeycrisp and *Rvi17* locus in Antonovka.

## Materials and methods

### Antonovka PacBio HiFi sequencing

Dormant branches of Antonovka 172670-B tree (PI 589956), an accession related to Antonovka Obyknovennaja, were collected from a research orchard at Cornell AgriTech, Geneva, NY, USA. These branches were placed in water until the leaves started to emerge and were subsequently kept in the dark for 2 days. After 2 days, the leaves were sampled and immediately frozen at −80°C. Frozen leaves were shipped on dry ice to the Genotyping Center of the University of Delaware, USA for high-molecular-weight genomic DNA extraction and Pacific BioSciences (PacBio) Single Molecule Real Time (SMRT) sequencing as previously described (Khan *et al.* 2022). For PacBio sequencing, the DNA was fragmented into 15-kb fragments, followed by construction of the HiFi library using the SMRTbell Express Template Prep Kit 2.0, and the DNA/Polymerase Binding Kit 2.0 (Pacific Biosciences), according to the manufacturer's protocol. The library was filtered for fragments >10 kb using Sage Blue Pippin (Sage Sciences) to remove smaller fragments and adapter dimers. PacBio Sequel IIe was used in the CCS/HiFi mode with a single SMRT cell with 2 h of pre-extension and 30-h movie times to sequence the library. Finally, the read length distribution and quality of the obtained reads were assessed using Pauvre v0.1923 (Schultz *et al.* 2018).

### Phased haplome assembly and scaffolding

Raw HiFi data were used to generate 21-mers to estimate the genome size and heterozygosity level using Jellyfish v2.3.0 (RRID:SCR_005491; Marçais and Kingsford 2011) and GenomeScope 2 (Ranallo-Benavidez *et al.* 2020). The HiFi reads were assembled into phased contigs using hifiasm v0.16.1 (RRID:SCR_021069; Cheng *et al.* 2021) and purged to remove any additional duplicates using purge_dups v1.2.6 (Guan *et al.* 2020). The mitochondrial and plastid genomes were assembled using the contig data by running the MitoHiFi function of MitoHiFi v3.0.0, and the corresponding redundant contigs of each individual haplome were filtered from the assembly (Uliano-Silva *et al.* 2004). Adapter and foreign DNA contaminations were detected and trimmed using NCBI Foreign Contamination Screening (FCS) pipeline fcs 0.3.0, i.e. FCS-adaptor and FCS-GX (Tatusova *et al.* 2016). The assembled haplotype-resolved contigs were scaffolded into phased chromosomes using the HaploSplit function of the HaploSync v1.0 package (Minio *et al.* 2022), by mapping the Antonovka contigs to the GDDH13 v1.1 reference genome assembly (Daccord *et al.* 2017). Genome quality and completeness were assessed using Benchmarking Universal Single-Copy Orthologs (BUSCO) v5.2.2 (RRID:SCR_015008; Manni *et al.* 2021) with the “eudicots_odb10” database. Haplome quality values (QVs) and *k*-mer completeness were assessed using Merqury v1.3 (Rhie *et al.* 2020). A synteny dotplot between the 2 haplomes was generated using MUMmer v4.0 (RRID:SCR_018171) (Kurtz *et al.* 2004) and Assemblytics (Nattestad and Schatz 2016).

### Repeat annotation and gene prediction

Apple repeat sequence libraries previously constructed from the genomes of “Gala,” *Malus sieversii*, and *Malus sylvestris* (Sun *et al.* 2020) were used to mask the Antonovka genome assembly. Redundant repeat sequences in these libraries were removed using the “cleanup_nested.pl” script in the EDTA package (v 2.1.0) with default parameters (Ou *et al.* 2019). The resulting nonredundant repeat library was used to mask each of the 2 Antonovka haplomes using RepeatMasker (v4.0.8; http://www.repeatmasker.org/). Protein-coding genes were predicted from the repeat-masked Antonovka genome assembly with MAKER (Cantarel *et al.* 2008), which integrates evidence from ab initio gene prediction, transcript, and protein evidence. AUGUSTUS (Stanke *et al.* 2006) and SNAP (Korf 2004) were used for ab initio gene predictions. Genome-guided transcript assembly of RNA-seq data reported by Sun *et al.* (2020) and CDS sequences from published apple genomes (Daccord *et al.* 2017; Zhang *et al.* 2019; Sun *et al.* 2020) were used as transcript evidence. Proteome sequences of published apple, peach, strawberry, and Arabidopsis genomes, as well as those from the UniProt database (Swiss-Prot plant division), were used as the protein homology evidence. To functionally annotate the predicted genes, their protein sequences were searched against the SwissProt and TrEMBL databases (https://www.uniprot.org/) using BLASTP with an e-value cutoff of 1E-5, and the InterPro database (https://www.ebi.ac.uk/interpro/) using InterProScan (Paysan-Lafosse *et al.* 2023).

### Genome sequence retrieval and identification of *HcrVf* homologs

Publicly available apple genome assemblies were downloaded from the Genome Database for Rosaceae (Jung *et al.* 2019) to identify *HcrVf1* and *HcrVf2* homologs in apple cultivars and wild *Malus* species. To estimate similarity levels among different *HcrVf* homologs from the *Rvi6* locus of *M. floribunda* 821, *HcrVf2* (GenBank identifier AJ297740) was compared against *HcrVf1*, *-3*, and *-4* (AJ297739, AJ297741, and EU794466, respectively; Supplementary Table 1). The assemblies of *M. domestica* cultivars Antonovka, Honeycrisp, HFTH1 (anther-derived homozygous line of “Hanfu” apple), “Golden Delicious” (GDDH13), “Gala,” and wild *Malus* species *M*alus *baccata*, *Malus prunifolia* Fupingqiuzi, *M. sieversii*, and *M. sylvestris* (Chen *et al.* 2019; Zhang *et al.* 2019; Sun *et al.* 2020; Khan *et al.* 2022; Li *et al.* 2022) were used to generate a BLAST database using the nucl function of the makeblastdb algorithm (Hahsler and Nagar 2019). Highly homologous sequences were identified by querying the coding sequences of *HcrVf1* and *HcrVf2* (AJ297739 and AJ297740, respectively) against the generated genomic database using the parameters of a maximal *e*-value of 1*e*−6, identity above 70%, and query coverage over 80%. The resulting blast output was transformed into FASTA files for comparative analyses.

#### Sequence alignment and phylogenetic analysis

Homologous sequences were aligned using ClustalW of the msa v1.30.1 multiple sequence aligner (Bodenhofer *et al.* 2015) in the R statistical environment to determine the sequence similarity among the identified homologs of *HcrVf1* and *HcrVf2*. The effects of differences and similarities among the aligned sequences were assessed and visualized using principal component analysis (PCA) using the glPca v2.1.8 function (Jombart and Ahmed 2011), which assigns loading scores to the variance of individual bases at different alignment positions. The degree of differences at individual positions was displayed by visualizing matches, mismatches and gaps, and estimating entropy among the homologs using the msavisr function of the seqvisr package v0.2.6 (Raghavan 2021) and seqdef of the TraMineR package v2.2-5 in combination with ggseqeplot v0.8.1, respectively (Gabadinho *et al.* 2011). Furthermore, neighbor-joining phylogenetic analysis was performed using MEGA11 (Tamura *et al.* 2021). First, the best-fitting statistical model was identified using the default settings of the Model Selection tool. The selected model was subsequently applied to construct a tree with 100 bootstrap replicates. The constructed phylogenetic trees were displayed using the Interactive Tree Of Life online tool (Letunic and Bork 2021).

#### Homolog mapping and haplotype analysis

Genome sequence of LGs harboring homologs with the highest similarity to *HcrVf2* was aligned to visualize syntenic regions and single nucleotide polymorphism (SNP) and gene density in the region of interest. Haplotypes of Antonovka (A), Honeycrisp (A and B), Gala (A), and *M. sieversii* (A) were aligned using minimap2 v2.24 (Li 2021). Pairwise bed alignment files showing structural variation and SNP positions were generated by SyRI v1.6.3 (Goel *et al.* 2019), and visualized by plotsr v1.1.1 (Goel and Schneeberger 2022). Antonovka gene positions were retrieved from the sequence annotation files.

The physical positions of the homologs were retrieved from BLAST results and visualized using the circlize v0.4.15 package (Gu *et al.* 2014) to determine the physical genomic positions of the identified homologs of *HcrVf2* and *HcrVf1* relative to the physical genomic positions of the microsatellite markers MdExp7, Ch-Vf1, and NzMS on LG 1 of apples. The physical genomic positions of these 3 markers were obtained by blasting their primer sequences (Clark 2014) against the respective apple genome assemblies.

To further verify the identity of the homologs and their identity by state or descent to the *HcrVf* genes, haplotype analysis was carried out using the publicly available 20K apple SNP array data (Bianco *et al.* 2014; Howard *et al.* 2021). Briefly, the accessions that have been genotyped were used to determine the numbers and lengths of shared haplotypes for evaluating their pedigree relationships. The 4 relationship groups consisted of accessions with available SNP data and a known relationship with a LG 1 apple scab-resistance genotypes (Supplementary Table 2 and File 1): (1) accessions harboring the *Rvi6* gene from *M. floribunda* 821 (“Liberty,” “Remo,” “Goldrush,” “Nova Easygro,” and “Topaz”), (2) Antonovka (Antonovka OB) harboring the *Rvi17* gene, (3) Honeycrisp harboring the *Vhc1* gene, and (4) scab-susceptible accessions (“McIntosh,” “Fuji,” “Idared,” Golden Delicious, and Gala; Howard *et al.* 2021). The SNP data set was used to parse the output from SPLoSH analysis (Howard *et al.* 2021) using the threshold of 4 cM and LG 1 data on the individuals that were visualized.

#### Protein domain analysis

Six selected amino acid sequences with the highest homology to *HcrVf2* and the corresponding *HcrVf1* homologs were searched against the InterPro database using InterProScan (Mulder and Apweiler 2007) with default parameters for protein domain identification. The identified domains of each homolog were visualized and aligned based on their position from the start of the sequence using the online InterProScan module (Blum *et al.* 2021).

## Results

### Haplotype-resolved chromosome-scale assembly of Antonovka

**Fig. 1.**
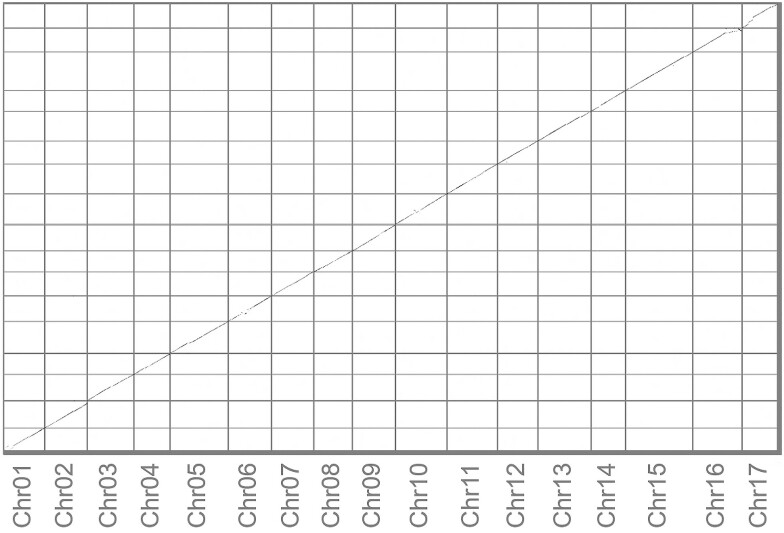
Mummer dotplot of haplomes A and B (*x* axis and *y* axis, respectively) of the Antonovka 172670-B apple.

In total, 932,852 PacBio HiFi reads with an average length of 16,150 bp were generated, and ∼90% of reads had lengths >11,000 bp, which resulted in a total of 15.1 Gb of reads, corresponding to ∼26× coverage of the Antonovka genome. The estimated genome size and heterozygosity level, based on 21-mers, were 530,399,965 bp and 1.38%, respectively. Two phased haplomes, haplome A (HAP1) and haplome B (HAP2), of Antonovka were de novo assembled into contigs, followed by chromosome assembly using the GDDH13 genome v1.1 as the reference (Daccord *et al.* 2017). Both haplomes were highly contiguous and similar in size (Table 1). HAP1 was 651 Mb in length, and contained 222 contigs with a contig N50 of 35.4 Mb, whereas HAP2 was 636 Mb in length, and contained 114 contigs with a contig N50 of 36.4 Mb (Table 1). The final HAP1 and HAP2 contained 17 chromosomes, with 97.3 and 97.8% of the assembled contig sequences in the pseudomolecules, respectively. High genome completeness for both haplomes was suggested based on Merqury *k*-mer and BUSCO analyses (Supplementary Fig. 1). HAP1 and HAP2 showed *k*-mer completeness of 78.2 and 77.7% with QVs of 59.2 and 59.3, respectively, yielding a total completeness of 96.5% (Table 1). The BUSCO completeness of HAP1 was 97.7% and that of HAP2 was 97.5% with high mutual structural similarity (Fig. 1). A total of 401.2 Mb (61.7%) and 390.8 Mb (61.4%) repetitive sequences were identified in HAP1 and HAP2, respectively, of the Antonovka genome (Supplementary Table 3). Furthermore, a total of 45,200 and 44,969 protein-coding genes were predicted for HAP1 and HAP2, respectively, with BUSCO completeness rates of 97.4 and 97.1%, respectively.

**Table 1.** Summary statistics for phased chromosome-level genome assembly of Antonovka apples.

*Assembly*	*Length (bp)*	*N*o. *of contigs*	*Longest contig (bp)*	*N50 (bp)*	*L50*	*QV*	*k-mer completeness (%)*	*BUSCO (%)*
*Antonovka* h*aplome A*	650,486,495	222	55,265,621	35,436,250	8	59.2	78.2	97.7
*Antonovka* h*aplome B*	636,545,088	114	54,180,258	36,356,697	8	59.3	77.7	97.5
*Combined*	1,287,031,583					59.3	96.5	

BUSCO, Benchmarking Universal Single-Copy Orthologs; bp, base pairs; QV, quality value.

### Homologs of HcrVf2 and HcrVf1 apple scab-resistance genes at Vhc1 and Rvi17 loci of Honeycrisp and Antonovka

This Antonovka assembly and previously published *Malus* genome assemblies enabled us to identify *HcrVf2* and *HcrVf1* candidate homologs in scab-resistant cultivars Antonovka, Honeycrisp, and other *Malus* genotypes without known scab resistance. Analysis of the DNA sequence of the *HcrVf2* homolog from the *Rvi6* locus of *M. floribunda* 821 (GenBank accession AJ297740) when compared with the *HcrVf1*, *-3*, and *-4* homologs (GenBank accessions AJ297739, AJ297741, and EU794466, respectively; Supplementary Table 1), revealed similar levels of identity, coverage, and *e*-values to *HcrVf2* for all 3 homologs (92–95%, 54–55%, and <1*e*−6, respectively). A total of 43 and 45 sequences homologous to the 2,943- and 3,048-bp coding regions of *HcrVf2* and *HcrVf1*, respectively, were identified in Antonovka, Honeycrisp, and other available apple genome assemblies (Table 2 and Supplementary Table 4). Identity and coverage for *HcrVf2* ranged between 90 and 95% (Table 2), and between 87–92% (identity) and 81–94% (coverage) for *HcrVf1* (Supplementary Table 4), respectively. The majority of these homologs, i.e. 24 and 38 sequences, respectively, were located on LG 1, while the remaining homologs were located on LG 6, 11, or 17, or were assigned to a scaffold. Homologous sequences with the highest identity and coverage of *HcrVf2*, the causative resistance gene of the *Rvi6* locus, were found on LG 1 of haplome A of the scab-resistant cultivars Honeycrisp and Antonovka. Both showed 2,801 (95%) identities and 6 gaps when aligned with *HcrVf2*. The overall blast score of 4,669 of these 2 homologs was followed by the scores of sequences identified in Honeycrisp haplome B and scab-susceptible cultivar Gala and *M. sieversii*, on LG 1B, 1A, and 11A, respectively. In contrast to the sequences with the highest identity and coverage to *HcrVf2*, *HcrVf1*-homologous sequences with the highest scores were identified in wild *Malus* species, i.e. *M. baccata*, *M. sieversii*, and *M. prunifolia*, followed by the sequences from Honeycrisp and Antonovka (Supplementary Table 4). The identity and coverage of the former 3 sequences did not exceed 92 and 94%, respectively, and contained more gaps, i.e. ranging from 60 to 87 gaps. In the latter 2 cultivars, they were even lower at 91 and 94%, respectively.

**Table 2.** Comparison of the identified *HcrVf2* homolog sequences across 9 genome assemblies of *M. domestica* and *Malus* species generated using BLAST (https://github.com/mhahsler/rBLAST).

Genotype	Haplome	NCBI accession number	Linkage group	Identities (bp)	Identity (%)	Gaps	Coverage (%)	Score	*e*-value	Start position (bp)	End position (bp)
**Honeycrisp**	A	PRJNA791346	1	2,801	95	6	95	4,669	0	28,925,028	28,927,964
				2,690	91	23	91	4,107	0	28,504,201	28,501,252
				2,635	89	36	90	3,904	0	28,457,974	28,455,056
**Antonovka**	A	PRJNA941883	1	2,801	95	6	95	4,669	0	28,917,218	28,920,154
				2,693	91	22	92	4,127	0	28,481,085	28,478,137
**Honeycrisp**	B	PRJNA791346	1	2,753	93	18	94	4,438	0	28,401,580	28,404,516
				2,697	92	51	92	4,209	0	28,468,462	28,471,348
				2,699	92	3	92	4,206	0	28,015,443	28,012,504
**Gala**	A	PRJNA591623	1	2,753	93	18	94	4,438	0	26,607,069	26,610,005
				2,697	92	51	92	4,209	0	26,680,477	26,683,363
***Malus sieversii***	A	PRJNA591623	11	2,755	93	21	94	4,420	0	6,460,988	6,463,933
***Malus sieversii***	A	PRJNA591623	1	2,743	93	24	93	4,419	0	28,679,434	28,682,347
				2,699	92	3	92	4,206	0	28,230,082	28,227,143
***Malus sylvestris***	B	PRJNA591623	1	2,755	93	36	94	4,412	0	26,578,480	26,581,416
				2,702	92	3	92	4,220	0	26,102,201	26,099,262
***Malus sylvestris***	A	PRJNA591623	1	2,754	93	36	94	4,407	0	27,348,207	27,351,143
				2,684	91	22	91	4,089	0	26,878,977	26,876,030
**HFTH1**	N/A	PRJNA482033	1	2,716	93	21	92	4,350	0	27,948,934	27,951,840
				2,688	91	22	91	4,104	0	27,497,327	27,494,379
**Golden Delicious**	/	PRJNA379390	1	2,716	93	21	92	4,350	0	27,934,209	27,937,115
				2,688	91	22	91	4,104	0	27,479,142	27,476,194
**Antonovka**	B	PRJNA941884	1	2,715	93	23	92	4,335	0	27,227,268	27,230,174
				2,688	91	22	91	4,104	0	26,775,597	26,772,649
**Gala**	B	PRJNA591623	1	2,699	92	3	92	4,206	0	23,724,870	23,721,931
***Malus prunifolia***	/	PRJNA354212	17	2,698	92	3	92	4,202	0	23,451,296	23,448,357
**Gala**	/	PRJNA591623	scf1458	2,698	92	3	92	4,202	0	9,353	6,414
***Malus prunifolia***	/	PRJNA354212	1	2,709	92	22	92	4,199	0	24,855,298	24,852,350
***Malus baccata***	/	PRJNA428857	scf292	2,678	91	24	91	4,079	0	121,680	118,744
***Malus sylvestris***	B	PRJNA591623	6	2,663	90	20	90	4,039	0	3,618,722	3,621,646
***Malus sieversii***	A	PRJNA591623	6	2,660	90	20	90	4,025	0	3,769,336	3,772,260
**Antonovka**	B	PRJNA941884	6	2,660	90	20	90	4,025	0	3,317,522	3,320,446
***Malus prunifolia***	/	PRJNA354212	6	2,659	90	20	90	4,020	0	3,996,785	3,999,709
				2,650	90	18	90	3,985	0	4,007,770	4,010,694
***Malus sieversii***	B	PRJNA591623	6	2,656	90	18	90	4,012	0	3,296,327	3,299,251
***Malus baccata***	/	PRJNA428857	scf243	2,650	90	18	90	3,985	0	2,081,416	2,084,340
**Honeycrisp**	B	PRJNA791346	6	2,650	90	20	90	3,980	0	3,264,718	3,267,642
**Golden Delicious**	/	PRJNA379390	6	2,649	90	20	90	3,975	0	3,273,930	3,276,854
**Gala**	B	PRJNA591623	6	2,649	90	20	90	3,975	0	3,376,658	3,379,582
**HFTH1**	/	PRJNA482033	6	2,647	90	18	90	3,972	0	3,296,664	3,299,588
**Gala**	A	PRJNA591623	6	2,647	90	18	90	3,972	0	4,060,224	4,063,148
**Honeycrisp**	A	PRJNA791346	6	2,644	90	20	90	3,953	0	3,526,083	3,529,007
**Antonovka**	A	PRJNA941883	6	2,644	90	20	90	3,953	0	3,430,807	3,433,731
***Malus sylvestris***	A	PRJNA591623	6	2,635	90	43	90	3,929	0	3,381,396	3,384,297

The scores show the comparison of a homolog with the *HcrVf2* coding sequence, and the position indicates its location in the corresponding genome.

bp, base pairs; NCBI, The National Center for Biotechnology Information.

**Fig. 2.**
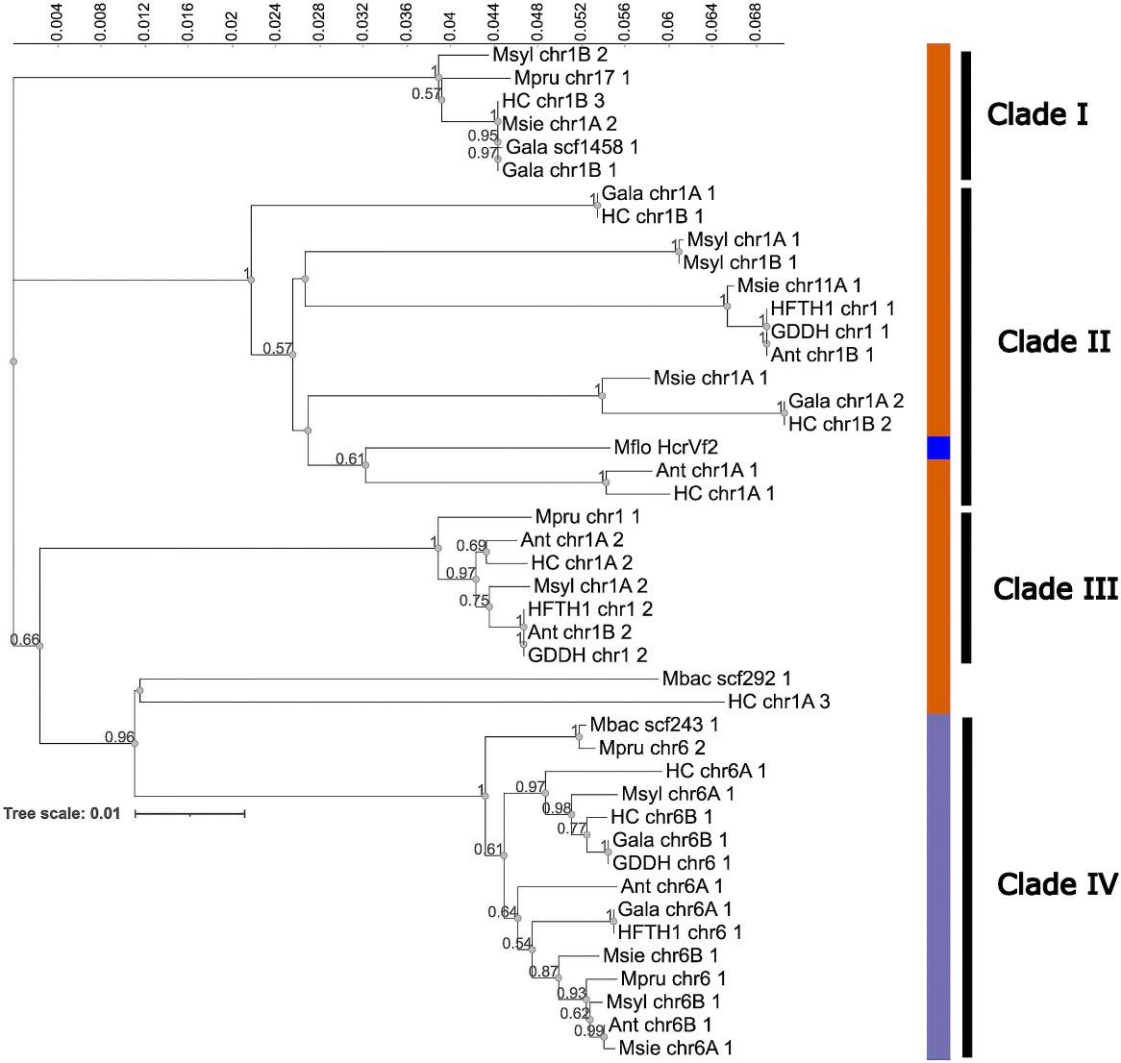
Phylogeny of *HcrVf2* homologs from 12 genome assemblies of *M. domestica* and wild *Malus* genotypes generated by neighbor-joining phylogenetic analysis using MEGA11 (Tamura *et al.* 2021). The 43 homologs of *HcrVf2* from *Malus* formed 4 major clades, supported by bootstrap values. The majority of members in clades I, II, and III were located on chromosome 1 or were placed to another LG, i.e. 11 or 17, or a scaffold (orange). Sequences from clade IV were found on LG 6 or on an unplaced scaffold (purple). Within clade II, *HcrVf2* from *M. floribunda* 821 can be found (blue). The first part of the gene name represents accession/cultivar name (HC, Honeycrisp; Ant, Antonovka 172670-B; Mflo, *M. floribunda* 821; Mbac, *M. baccata*; Mpru, *M. prunifolia*; Msyl, *M. sylv*e*stris*; Msie, *M. sieversii*; HFTH1, anther-derived homozygous genotype HFTH1; GDDH13, doubled-haploid derivative of Golden Delicious), followed by a chromosome number, haplome (if available), and the ranking based on the blast score from the same genotype. Numbers on nodes are bootstrap values, and values <0.50 are not shown.

Neighbor-joining phylogenetic analysis of the aligned homologous sequences confirmed the BLAST results and grouped the sequences into 4 major clades (Fig. 2). Sequences found in the second clade, i.e. those on LG 1, showed the highest relatedness to *HcrVf2* of *M. floribunda* 821. In particular, the haplome A homologs of scab-resistant genotypes Antonovka and Honeycrisp showed the highest relatedness to *HcrVf2*. They showed a moderate node support with a bootstrap value of 0.61 compared with *HcrVf2* and a maximum node support with a bootstrap value of 1. This clade showed a lower node support with the most closely related clade, i.e. a bootstrap value <0.50, which comprised sequences on LG 1 of Honeycrisp haplome B, scab-susceptible cultivar Gala haplome A and *M. sieversii* haplome A. Other *HcrVf2* homologs of the second clade were more distant from *HcrVf2* and exceeded the node-support bootstrap value of 0.57, or even belonged to a different clade. Similarly, the PCA (Supplementary Fig. 2) showed that sequences from LG 1 formed a tight cluster around *HcrVf2* with positive scores along components 1 and 2, which explained 21.6 and 13.1% of the total sequence variability, respectively. Indeed, the cluster contained abovementioned sequences from Antonovka, Honeycrisp, Gala, and *M. sieversii.* The underlying sequence variation is illustrated by the increased sequence entropy along the entire sequence, and with the highest increase at the position between 2,000 and 2,500 bp (Supplementary Fig. 3).

In contrast to *HcrVf2*, neighbor-joining phylogenetic analysis of *HcrVf1* homologs from scab-resistant and susceptible/wild *Malus* accessions indicated that the sequences found in wild *Malus* accessions were the most related to *HcrVf1* (Supplementary Fig. 4). The sequences were grouped into 4 clades. Clade IV contained *HcrVf1* and its most related homologs. Among these, homologs on LG 1 of *M. baccata* and *M. sieversii* showed the highest relatedness to *HcrVf1*, with strong node support (bootstrap value of 0.89), followed by the homologs from LG 1 of *M. prunifolia* and *M. baccata* scaffold 241, which showed low node support (bootstrap value <0.50). Other homologs found in clade IV, including those from haplotome A of LG 1 of scab-resistant Honeycrisp and Antonovka, showed lower relatedness with strong node-support bootstrap values of 1. The other clades were even more distant from *HcrVf1*, with the maximum node-support bootstrap values of 1. PCA based on sequence similarity (Supplementary Fig. 5) showed that the sequences from LG 1 formed a tight cluster around the *HcrVf1*. Sequences from this cluster showed negative and positive scores along principal components 1 and 2, explaining 15.9 and 13.8% of the total sequence variability, respectively. The underlying sequence variation is depicted by increased sequence entropy along the entire sequence, with the peak entropy at a position of approximately 2,500 bp (Supplementary Fig. 6).

Finally, phylogenetic analysis of only one region within the *HcrVf2* sequences, i.e. the 700–800-bp long PCR products of scab-resistant or scab-susceptible genotypes (Boudichevskaia *et al.* 2009), was unable to confirm observations based on the entire gene (Supplementary Fig. 7). PCR products of “Releta,” “Regia” B, Regia A (non-*Rvi6* accessions), and “Prima” (*Rvi6* accession) showed the highest similarity to *Hcrvf2*, while homologs from scab-resistant Honeycrisp and Antonovka LG 1 of haplome A showed a low relatedness to the analyzed region of *HcrVf2*.

### Location of *HcrVf* homologs and haplotype identity of LG 1 in scab-resistant and susceptible genotypes

**Fig. 3.**
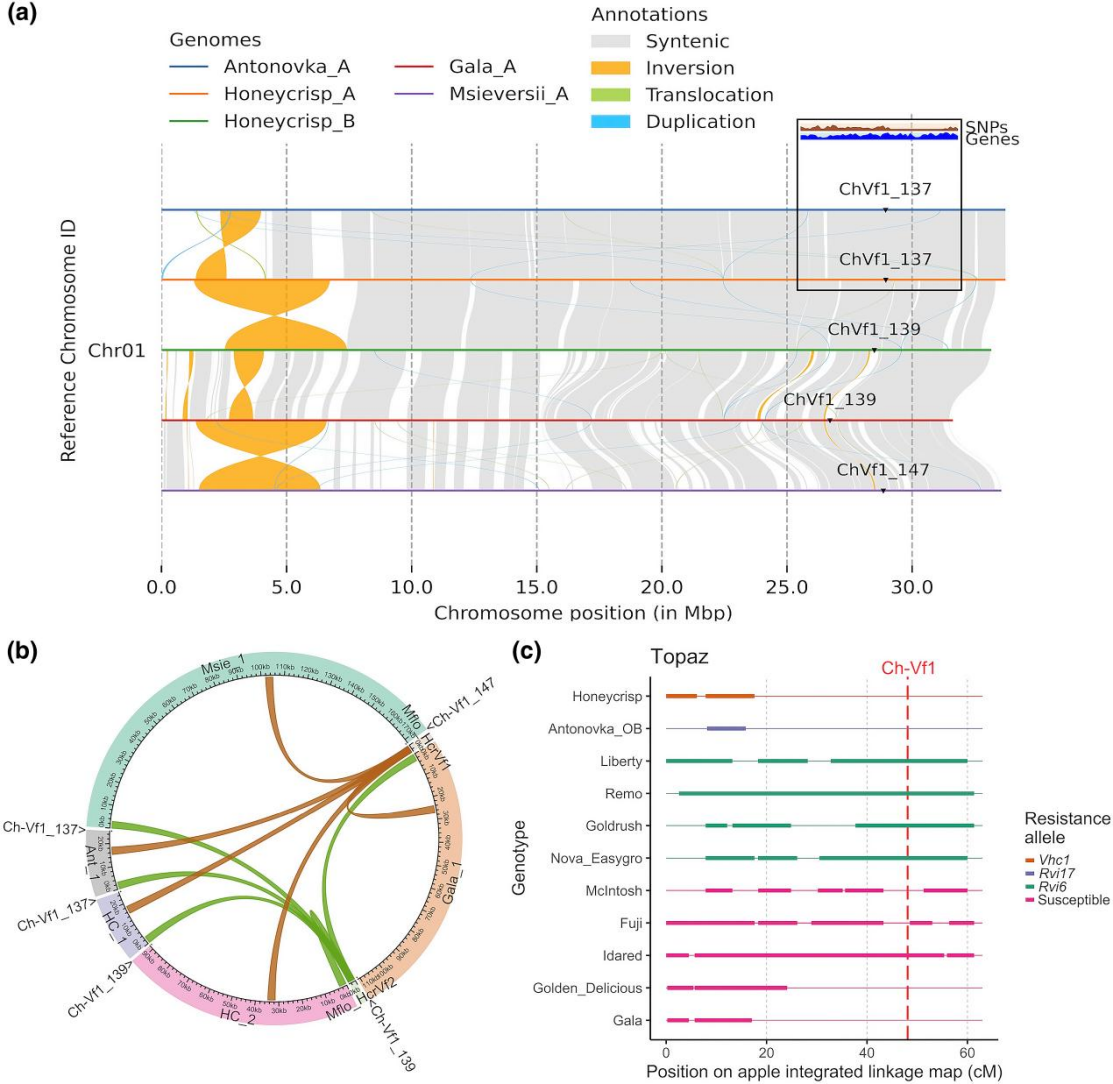
Visualization of the synteny, location of *HcrVf* homologs, and shared SNPs on LG 1. a) Synteny plot of the LG 1 from Antonovka haplome A, Honeycrisp haplomes A and B, Gala haplome A, and *M. sieversii* haplome A, indicating the position and size of the Ch-Vf1 scab-resistance marker, and SNP and gene density along the up- and downstream flanking regions. b) Circoplot showing the physical positions of the most related *HcrVf2* (green lines) and *HcrVf1* (brown lines) homologs in the respective genome assemblies of haplome A of Honeycrisp and Antonovka, Gala, haplome B of Honeycrisp, and *M. sieversii* relative to the location of the Ch-Vf1 microsatellite marker. c) Extended shared haplotypes of the SNP marker data across the LG 1 from *Rvi6*-harboring Topaz with accessions known to carry different resistances, i.e. *Rvi6* from *M. floribunda* 821, *Vhc1* from Honeycrisp, *Rvi17* present in Antonovka, or those found to be susceptible to the majority of *V. inaequalis* races. Thin and bold lines indicate SNP allelic regions that are not shared/identical and shared/identical, respectively, with the SNPs in Topaz. The red dashed vertical lines indicate the approximate location of the Ch-Vf1 marker on the genetic map.

Synteny analysis of the haplomes harboring *HcrVf2* and *HcrVf1* homologs with the highest similarity to *HcrVf2* showed synteny between the scab-resistance regions of haplome A from Antonovka and haplomes A and B from Honeycrisp (Fig. 3a). Synteny was observed on both flanking sides of the Ch-Vf1 scab-resistance marker between haplomes A of Antonovka and Honeycrisp. The SNP density was higher upstream from the marker compared with the downstream region. In fact, synteny was observed between all haplotypes from Antonovka and Honeycrisp (Supplementary Fig. 8). In contrast, no synteny was observed in the Ch-Vf1 flanking regions between these haplomes and the haplomes A from scab-scuseptible Gala and *M. sieversii*. The latter 2 haplotypes showed synteny in the Ch-Vf1 flanking regions.

Mapping of the *HcrVf2* and *HcrVf1* homologs on LG 1 revealed that the positions of the most related homologs from scab-resistant Antonovka and Honeycrisp haplome A shared the same physical location (Fig. 3b and Supplementary Fig. 9). The location of the homologs in these 2 cultivars was 23–25 kb away from the 137-bp Ch-Vf1 marker, i.e. the marker used to determine the scab resistance of *Rvi6*-harboring genotypes. In contrast, other homologs in these 2 cultivars were more distant from Ch-Vf1, with distances of over 400 kb. Distances of the most related homologs from scab-susceptible Gala and the haplome B of Honeycrisp were even more distant, i.e. >1.68 Mb from the 139-bp Ch-Vf1 marker. A similarly large distance was observed between the marker and the most closely related homolog found in *M. sieversii* haplome A.

Furthermore, Honeycrisp and Antonovka (Common Antonovka) shared resistance haplotypes near the Ch-Vf1 marker, while their haplotypes at this locus differed from those of cultivars harboring resistance from *M. floribunda* 821 (Fig. 3c and Supplementary Fig. 10). Shared haplotype analysis of this region based on the SNPs of genotypes harboring *Rvi6*, *Vhc1*, *Rvi17*, or no resistance loci, indicated that *HcrVf2*-containing cultivars, i.e. Liberty, Remo, Goldrush, and Nova Easygro, showed a large shared genomic region with Topaz in proximity of Ch-Vf1. In contrast, no similarities were observed between SNPs in the Ch-Vf1 region of Antonovka and Honeycrisp compared with Topaz (Fig. 3c). However, haplomes of Honeycrisp and Antonovka, which are thought to harbor resistance alleles, share the distal part of LG 1 near Ch-Vf1 (Supplementary Fig. 10). Finally, scab-susceptible McIntosh, Fuji, Golden Delicious, and Gala did not show SNP identity with Topaz in the close proximity of Ch-Vf1 (Fig. 3c), whereas Idared showed large parts of the LG 1 as shared with Topaz. Ch-Vf1 regions of Golden Delicious, Idared, and Gala harbored SNPs that are mostly shared with Honeycrisp (Supplementary Fig. 10).

### Amino acid sequence and protein domains of *HcrVf* homologs in scab-resistant genotypes

Amino acid sequence alignment and neighbor-joining phylogenetic analysis of HcrVf2 confirmed the observations at the DNA level (Supplementary Figs. 11 and 12). Homologs from LG 1 of haplome A of scab-resistant cultivars Honeycrisp and Antonovka to HcrVf2 were the most closely related homologs, followed by those from LG 1 of Honeycrisp haplome B, the scab-susceptible cultivar Gala haplome A, and *M. sieversii* haplome A (Supplementary Fig. 11). Furthermore, different groupings of the sequences originating from LG 1 and 6 were observed at the amino acid level. In contrast to HcrVf2 homologs, amino acid analysis of HcrVf1 homologs revealed a more distinct phylogeny than that at the DNA level. In particular, neighbor-joining analysis indicated that sequences on LG 1 of *M. prunifolia* and scaffold 241 of *M. baccata* were the most similar to HcrVf1 (Supplementary Fig. 12). However, most of the homologs showed a greater distance from HcrVf1, with highly variable node-support bootstrap values.

**Fig. 4.**
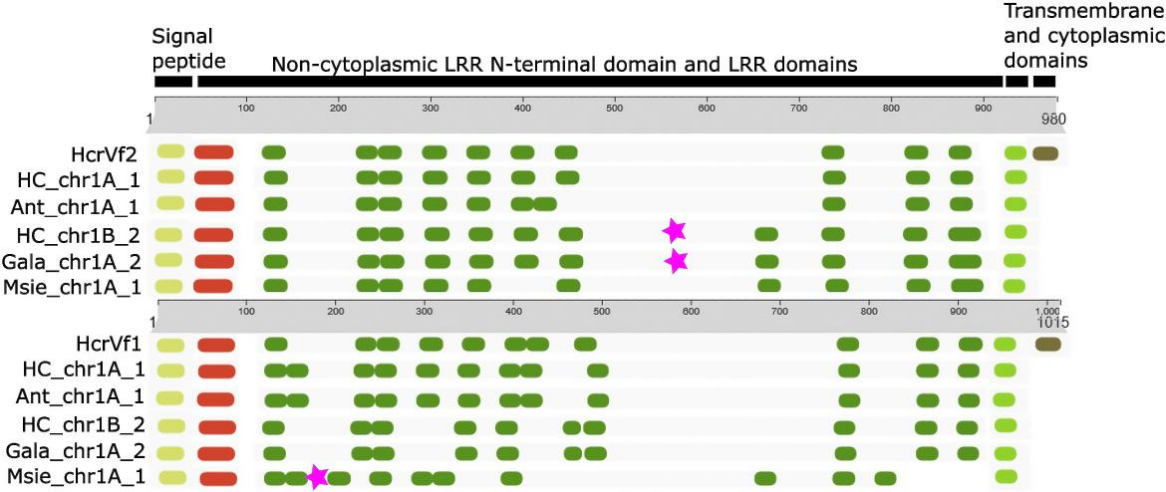
Schematic representations of the InterPro (Mulder and Apweiler 2007) domains of HcrVf2 and HcrVf1 homologs on LG1. Protein domains of the homologs with the highest relatedness to HcrVf2 and HcrVf1 (980 and 1015 amino acids in length, respectively) in scab-resistant accessions of *M. floribunda*, haplome A of Honeycrisp (HC) and Antonovka 172670-B (Ant), Gala, haplome B of Honeycrisp, and *M. sieversii* (Msie) include the signal peptide (yellow), N-terminus of the LRR region (red), extracellular LRR domains (dark green), hydrophobic transmembrane domain (light green), and the cytoplasmic terminus domain (brown). Stars indicate the presence of putative stop codons. The first part of the identification name represents accession/cultivar name followed by chromosome (chr) number and haplome (if available).

Protein domain analysis using InterPro of the homologs with the highest similarity to HcrVf2 in scab-resistant cultivars Honeycrisp and Antonovka, scab-susceptible cultivar Gala, and wild accession *M. sieversii* showed that the sequences from haplome A of Honeycrisp and Antonovka contained the same number of LRRs as HcrVf2 (Fig. 4). In addition, the positions of the majority of these LRRs overlapped with the HcrVf2 domains and contained the signal peptide, the noncytoplasmic domain containing LRRs, and the transmembrane domain. In Antonovka, the eighth LRR domain was found upstream the coding sequence compared with HcrVf2. In addition, the last cytoplasmic domain was absent in all the homologs. Haplotypes of Gala and haplome B of Honeycrisp contained a stop codon, which could result in a prematurely terminated protein. Finally, the wild accession *M. sieversii* showed the absence of an LRR domain and an extended LRR domain compared with HcrVf2. The signal peptide region, as well as several LRRs, showed specific amino acid differences between HcrVf2 and its homologs from Honeycrisp and Antonovka (e.g. positions 9, 17, 26, etc.; Supplementary Fig. 13). Occasionally, other 3 sequences from Honeycrisp haplome B, Gala, and wild accession *M. sieversii* showed additional changes in amino acids compared with HcrVf2 (e.g. positions 93, 94, and 96–100).

All HcrVf1 homologs showed variable number and position of LRR domains, with an absence of the cytoplasmic domain (Fig. 4). The most similar homologs from scab-resistant cultivars, i.e. Honeycrisp and Antonovka, showed an additional third LRR domain, whereas the homologs of Gala and haplome B of Honeycrisp showed an absence of the fifth domain. The homolog from *M. sieversii* contained a stop codon after the third LRR domain. A high number of insertions and amino acid polymorphisms were found among all the HcrVf1 homologs (Supplementary Fig. 14).

## Discussion

We assembled a high-quality phased chromosome-scale genome of the Antonovka 172670-B apple with a BUSCO completeness of over 97.5%. The quality of the Antonovka genome assembly is comparable with that of Honeycrisp, GDDH13, and Gala genomes with BUSCO completeness rates of 98.7, 97.4, and 97.7%, respectively (Daccord *et al.* 2017; Sun *et al.* 2020; Khan *et al.* 2022). The high quality and phased nature of this genome make it relevant for the genetic identification and characterization of allelic variations in highly heterozygous plants (Khan and Korban 2022). Phased genome assemblies with similarly high-quality haplomes have enabled identification of alleles linked with a variety of traits in domesticated apple cultivars and their wild progenitor species, as well as with other commercially important crops (Sun *et al.* 2020; Frommer *et al.* 2022; Hoopes *et al.* 2022). For example, using phased assemblies of Gala, *M. sieversii*, and *M. sylvestris* apples, allele-specific expression of *MYB1* and consequently the yellowish-red fruit skin color were found in Gala and *M. sieversii*. In addition, the “A” allele at base 1,455 of the *Ma1* coding gene sequence was found to be associated with the low fruit acidity of the former 2 genotypes. Indeed, the new Antonovka genome assembly in combination with the Honeycrisp assembly and other available *Malus* assemblies (Chen *et al.* 2019; Zhang *et al.* 2019; Sun *et al.* 2020; Khan *et al.* 2022; Li *et al.* 2022) enabled us to identify homologs of *HcrVf2* and *HcrVf1* on LG 1 and 6 across 9 analyzed genomes. These regions harbor dozens of resistance gene analogs, as demonstrated for LG 1 and 6 (Perazzolli *et al.* 2014; Singh *et al.* 2021). The identification of resistance gene homologues within a resistance locus can complement traditional association mapping, which often fails to pinpoint a set of high-confidence resistance gene candidates that can feasibly be functionally characterized (Dunemann and Egerer 2010; Clark 2014). The identified associated resistance loci on LG 1 of Honeycrisp and Antonovka were large spanning several Mb/cM, that is, 19 and 15 cM for *Vhc1* and *Rvi17*, respectively (Dunemann and Egerer 2010; Clark 2014). As a result, only 2 of the 19 major scab-resistance genes, i.e. *Rvi6* and *Rvi15*, have been functionally validated to date (Bus *et al.* 2011). Also, the new genome may enable further identification of additional alleles associated with other traits of Antonovka accessions including its unsurpassed strong and pleasant fruit aroma, cold hardiness, and disease resistance (Wilner 1960; Bus *et al.* 2012).

Among the identified homologs, the 2 sequences on LG 1 of Honeycrisp and Antonovka haplome A showed the highest relatedness to *HcrVf2*, the functional allele of the *Rvi6-*resistant locus. Sequence alignment analysis showed that these 2 sequences were not identical to *HcrVf2* but differed in several single nucleotides and in a deletion. This observation supports previous comparisons of alleles in these cultivars, showing that these genotypes contain different Ch-Vf1 marker alleles on the distal end of LG 1 (Bus *et al.* 2012; Clark 2014). Honeycrisp and Antonovka contain the marker allele that is 137 bp long (reported as a 139-bp allele), as opposed to the 159-bp allele associated with *HcrVf2* from *M. floribunda* 821. Furthermore, Honeycrisp and several Antonovka accessions showed resistance to different *V. inaequalis* isolates compared with the cultivars with *HcrVf2* (Bus *et al.* 2012). Unlike *HcrVf2*, which has been overcome in many apple-producing regions globally by *V. inaequalis* race 6 (Papp *et al.* 2019; Patocchi *et al.* 2020), *Rvi17* has been suggested to confer resistance to race 6 (Parisi and Lespinasse 1996). Defense responses of the *Vhc1* locus of Honeycrisp to a broad range of the US *V. inaequalis* strains are less well-defined and range from no symptoms to sporulation (Clark 2014).

Synteny, physical mapping, and haplotype sharing analyses of LG 1 demonstrated that the homologs in Honeycrisp and Antonovka with high similarity to *HcrVf2* and *HcrVf1* share physical location but are not identical by state (IBS) or descent (IBD) to *HcrVf* genes. In Honeycrisp and Antonovka, the flanking region of the Ch-Vf1 marker is highly syntenic among each other and the identified homologs are in tight physical proximity (∼20 kb) to the Ch-Vf1 marker. The locations of the identified homologs in these 2 cultivars may overlap with the locations of *HcrVf2* and *HcrVf1*, which have been identified to be <140 kb from the marker (Patocchi *et al.* 1993; Vinatzer *et al.* 2001, 2004). Other homologs were >400 kb apart from Ch-Vf1. Furthermore, no SNPs identical to those in *Rvi6*-harboring cultivars were identified across the region harboring the Ch-Vf1 marker in Honeycrisp and Antonovka, most likely rendering these regions different by state and descent. This is in line with previous observations that Antonovka and Honeycrisp contain different Ch-Vf1 alleles than *M. floribunda* and its related cultivars (Bus *et al.* 2012; Clark 2014). In contrast, haplomes A of Honeycrisp and Antonovka, as well as other Honeycrisp- and Antonovka-related cultivars, are syntenic and share extended haplotypes in the proximity of Ch-Vf1, suggesting that their homologs tightly linked to Ch-Vf1 could potentially be IBS. However, as no common ancestry of Antonovka and Honeycrisp is known, they show high SNP frequency upstream from Ch-Vf1, and because the shared haplotype mainly extend downstream the Ch-Vf1 over the region containing *HcrVf*, their IBD remains to be clarified. The extended shared haplotypes SNP data is made publicly accessible for use in breeding and genetic research (Supplementary File 1).

The nucleotide differences among the homologs with the highest similarity to *HcrVf2* and *HcrVf1* resulted in amino acid changes that were reflected in the number and size of the predicted protein domains. The 2 sequences on LG 1 of Honeycrisp and Antonovka haplome A showed only minor changes in the number and size of the domains, i.e. the lack of a cytoplasmic domain and a shift in 1 LRR domain in Antonovka. The high similarity (>92%) between the 2 sequences exceeded the relatedness of the 4 *Rvi6* locus paralogs, as HcrVf1 and HcrVf2 differed in the total number of LRR domains and showed lower identity (78–84%; Belfanti *et al.* 2004; Xu and Korban 2004). Similarly, in other species, the identity among resistance homologs, such as, *R2* in *Solanum*, ranged between 85 and 99% (Srivastava *et al.* 2018). However, larger changes in amino acid sequences and protein domains were observed in the homologs from Gala, Honeycrisp haplome B, and *M. sieversii*. In these homologs, either the presence of stop codons is predicted to result in premature proteins or they contain different numbers and positions of the protein domains. This resembles the *HcrVf3* gene in *M. floribunda* 821, compared with the other 3 *HcrVf* paralogs (Xu and Korban 2004, 2002)*. HcrVf3* contains a stop codon because of a single-point mutation at position 229, which most likely results in a prematurely terminated protein. Altogether, the presence or absence of the identified protein domains in different genotypes should be further validated for their functional roles in scab resistance.

In summary, construction of a novel phased genome assembly of scab-resistant Antonovka 172670-B and utilization of other available scab-resistant, scab-susceptible, and wild apple assemblies resulted in revealing candidate resistance genes with high homology to *HcrVf2* and *HcrVf1*. In scab-resistant genotypes Antonovka and Honeycrisp, 2 homologs on LG 1 of haplome A showed particularly high similarity and coverage to *HcrVf2*, the apple scab-resistance paralog of the *Rvi6* locus. These 2 homologs show high similarity to *HcrVf2* at the amino acid level, have minor changes in their protein motifs, and are located in close proximity to the Ch-Vf1 scab-resistance marker. Potentially, these 2 homologs are identical-by-state, as both homologs share extended syntenic haplotypes in the proximity of Ch-Vf1 and show an absence of shared SNPs with *M. floribunda* 821-related cultivars. Homologs identified in scab-susceptible Gala, haplome B of Honeycrisp and *M. sieversii* have a lower homology with *HcrVf2*. Furthermore, 2 homologs highly related to *HcrVf1* were identified in close proximity to the *HcrVf2* homologs on LG 1 of haplome A of Antonovka and Honeycrisp. These homologs show high sequence and protein domain variability. In fact, *HcrVf1* showed the highest relatedness to homologs identified in the wild *Malus* accessions. As a finality, the Antonovka 172670-B genome adds to the limited genomic resources comprising only partial coding DNA sequences of *HcrVf2* homologs and marker data associated with the resistance, and hence makes future comparison of the genetic basis for scab resistance and other beneficial traits of this cultivar more feasible. Information on putative resistance gene candidates for *Vhc1* and *Rvi17* genes in Honeycrisp and Antonovka can be utilized for further dedicated studies on functional validation and breeding of these homologs.

## Supplementary Material

jkad253_Supplementary_Data

## Data Availability

This Whole-Genome Shotgun project has been deposited at GenBank under the accession numbers JASJOP000000000 and JASJOQ000000000. The Antonovka genome raw sequencing reads have been deposited in NCBI BioProject database under accessions PRJNA941884 and PRJNA941883. Genome assembly and annotations are also available at Figshare (https://doi.org/10.6084/m9.figshare.23631369). Supplemental material available at G3 online.

## References

[jkad253-B1] Aranzana MJ , Decroocq V, Dirlewanger E, Eduardo I, Gao ZS, Gasic K, Iezzoni A, Jung S, Peace C, Prieto H, et al 2019. *Prunus genetics* and applications after *de novo* genome sequencing: achievements and prospects. Hortic Res. 6(1):58. doi:10.1038/s41438-019-0140-8. 30962943 PMC6450939

[jkad253-B2] Bastiaanse H , Bassett HCM, Kirk C, Gardiner SE, Deng C, Groenworld R, Chagné D, Bus VGM. 2016. Scab resistance in “Geneva” apple is conditioned by a resistance gene cluster with complex genetic control. Mol Plant Pathol. 17(2):159–172. doi:10.1111/mpp.12269. 25892110 PMC6638522

[jkad253-B3] Beckerman JL , Sundin GW, Rosenberger DA. 2013. Do some IPM concepts contribute to the development of fungicide resistance? Lessons learned from the apple scab pathosystem in the United States. Pest Manag Sci. 71(3):331–342. doi:10.1002/ps.3715. 24375947

[jkad253-B4] Belfanti E , Silfverberg-Dilworth E, Tartarini S, Patocchi A, Barbieri M, Zhu J, Vinatzer B, Gianfranceschi L, Gessler C, Sansavini S. 2004. The *HcrVf2* gene from a wild apple confers scab resistance to a transgenic cultivated variety. Proc Natl Acad Sci U S A. 101(3):886–890. doi:10.1073/pnas.0304808101. 14715897 PMC321776

[jkad253-B5] Bianco L , Cestaro A, Sargent DJ, Banchi E, Derdak S, Di Guardo M, Salvi S, Jansen J, Viola R, Gut I, et al 2014. Development and validation of a 20K single nucleotide polymorphism (SNP) whole genome genotyping array for apple (*Malus × domestica* Borkh). PLoS One. 9(10):e110377. doi:10.1371/journal.pone.0110377. 25303088 PMC4193858

[jkad253-B6] Blum M , Chang H-Y, Chuguransky S, Grego T, Kandasaamy S, Mitchell A, Nuka G, Paysan-Lafosse T, Qureshi M, Raj S. 2021, et al The InterPro protein families and domains database: 20 years on. Nucleic Acids Res. 49(D1):D344–D354. doi:10.1093/nar/gkaa977. 33156333 PMC7778928

[jkad253-B7] Bodenhofer U , Bonatesta E, Horejš-Kainrath C, Hochreiter S. 2015. msa: an R package for multiple sequence alignment. Bioinformatics. 31(24):3997–3999. doi:10.1093/bioinformatics/btv494. 26315911

[jkad253-B8] Boudichevskaia A , Flachowsky H, Dunemann F. 2009. Identification and molecular analysis of candidate genes homologous to HcrVf genes for scab resistance in apple. Plant Breed. 128(1):84–91. doi:10.1111/j.1439-0523.2008.01537.x.

[jkad253-B9] Broggini GAL , Galli P, Parravicini G, Gianfranceschi L, Gessler C, Patocchi A. 2009. *Hcrvf* paralogs are present on linkage groups 1 and 6 of *Malus*. Genome. 52(2):129–138. doi:10.1139/g08-115. 19234561

[jkad253-B10] Bus VGM , Rikkerink EHA, Caffier V, Durel C-E, Plummer KM. 2011. Revision of the nomenclature of the differential host-pathogen interactions of *Venturia inaequalis* and *Malus*. Annu Rev Phytopathol. 49(1):391–413. doi:10.1146/annurev-phyto-072910-095339. 21599495

[jkad253-B11] Bus VGM , van de Weg WE, Peil A, Dunemann F, Zini E, Forsline PL, Laurens FND, Blažek J, Hanke V, Forsline PL. 2012. The role of Schmidt ‘Antonovka’ in apple scab resistance breeding. Tree Genet Genomes. 8(4):627–642. doi:10.1007/s11295-012-0470-2.

[jkad253-B13] Calenge F , Faure A, Goerre M, Gebhardt C, van de Weg WE, Parisi L, Durel C-E. 2004. Quantitative trait loci (QTL) analysis reveals both broad-spectrum and isolate-specific QTL for scab resistance in an apple progeny challenged with eight isolates of *Venturia inaequalis*. Phytopathology. 94(4):370–379. doi:10.1094/PHYTO.2004.94.4.370. 18944113

[jkad253-B14] Cantarel BL , Korf I, Robb SMC, Parra G, Ross E, Moore B, Holt C, Sánchez Alvarado A, Yandell M. 2008. MAKER: an easy-to-use annotation pipeline designed for emerging model organism genomes. Genome Res. 18(1):188–196. doi:10.1101/gr.6743907. 18025269 PMC2134774

[jkad253-B15] Chen X , Li S, Zhang D, Han M, Jin X, Zhao C, Wang S, Xing L, Ma J, Ji J, et al 2019. Sequencing of a wild apple (*Malus baccata*) genome unravels the differences between cultivated and wild apple species regarding disease resistance and cold tolerance. G3 (Bethesda). 9(7):2051–2060. doi:10.1534/g3.119.400245. 31126974 PMC6643876

[jkad253-B16] Cheng H , Concepcion GT, Feng X, Zhang H, Li H. 2021. Haplotype-resolved *de novo* assembly using phased assembly graphs with hifiasm. Nat Methods. 18(2):170–175. doi:10.1038/s41592-020-01056-5. 33526886 PMC7961889

[jkad253-B17] Clark MD . 2014. Characterizing the Host Response and Genetic Control in “Honeycrisp” to Apple Scab (Venturia inaequalis). University of Minnesota Digital Conservancy, University of Minnesota. https://conservancy.umn.edu/handle/11299/172140.

[jkad253-B18] Clark MD , Bus VGM, Luby JJ, Bradeen JM. 2014. Characterization of the defence response to *Venturia inaequalis* in ‘Honeycrisp’ apple, its ancestors, and progeny. Eur J Plant Pathol. 140(1):69–81. doi:10.1007/s10658-014-0444-3.

[jkad253-B19] Cova V , Lasserre-Zuber P, Piazza S, Cestaro A, Velasco R, Durel CE, Malnoy M. 2015. High-resolution genetic and physical map of the *Rvi1 (Vg*) apple scab resistance locus. Mol Breed. 35(1):1–13. doi:10.1007/s11032-015-0245-1.

[jkad253-B20] Daccord N , Celton J-M, Linsmith G, Becker C, Choisne N, Schijlen E, van de Geest H, Bianco L, Micheletti D, Velasco R, et al 2017. High-quality *de novo* assembly of the apple genome and methylome dynamics of early fruit development. Nat Genet. 49(7):1099–1106. doi:10.1038/ng.3886. 28581499

[jkad253-B21] Dunemann F , Egerer J. 2010. A major resistance gene from Russian apple “Antonovka” conferring field immunity against apple scab is closely linked to the *Vf* locus. Tree Genet Genomes. 6(5):627–633. doi:10.1007/s11295-010-0278-x.

[jkad253-B22] FAOSTAT . 2021. FAOSTAT, Division, Food and Agriculture Organization of the United Nations. Statistics [WWW Document]. Available from https://www.fao.org/faostat/.

[jkad253-B23] Frommer B , Hausmann L, Holtgräwe D, Viehöver P, Hüttel B, Reinhardt R, Töpfer R, Weisshaar B. 2022. A fully phased interspecific grapevine rootstock genome sequence representing V. riparia and V. cinerea and allele-aware annotation of the phylloxera resistance locus Rdv1. bioRxiv 499180. 10.1101/2022.07.07.499180, preprint: not peer reviewed. PMC7380517 32273371

[jkad253-B24] Frommer B , Müllner S, Holtgräwe D, Viehöver P, Hüttel B, Töpfer R, Weisshaar B, Zyprian E. 2023. Phased grapevine genome sequence of an *Rpv12* carrier for biotechnological exploration of resistance to *Plasmopara viticola*. Front Plant Sci. 14:1180982. doi:10.3389/fpls.2023.1180982. 37223784 PMC10200900

[jkad253-B25] Gabadinho A , Ritschard G, Müller NS, Studer M. 2011. Analyzing and visualizing state sequences in R with TraMineR. J Stat Softw. 40(4):1–37. doi:10.18637/jss.v040.i04.

[jkad253-B26] Galli P , Patocchi A, Broggini GAL, Gessler C. 2010. The *Rvi15 (Vr2*) apple scab resistance locus contains three TIR-NBS-LRR genes. Mol Plant Microbe Interact. 23(5):608–617. doi:10.1094/MPMI-23-5-0608. 20367469

[jkad253-B27] Goel M , Schneeberger K. 2022. plotsr: visualizing structural similarities and rearrangements between multiple genomes. Bioinformatics. 38(10):2922–2926.doi: 10.1093/bioinformatics/btac196. 35561173 PMC9113368

[jkad253-B28] Goel M , Sun H, Jiao W-B, Schneeberger K. 2019. SyRI: finding genomic rearrangements and local sequence differences from whole-genome assemblies. Genome Biol. 20(1):277. doi:10.1186/s13059-019-1911-0. 31842948 PMC6913012

[jkad253-B29] Gu Z , Gu L, Eils R, Schlesner M, Brors B. 2014. Circlize implements and enhances circular visualization in R. Bioinformatics. 30(19):2811–2812. doi:10.1093/bioinformatics/btu393. 24930139

[jkad253-B30] Guan D , McCarthy SA, Wood J, Howe K, Wang Y, Durbin R. 2020. Identifying and removing haplotypic duplication in primary genome assemblies. Bioinformatics. 36(9):2896–2898. doi:10.1093/bioinformatics/btaa025. 31971576 PMC7203741

[jkad253-B31] Hahsler M , Nagar A. 2019. rBLAST: R interface for the basic local alignment search tool [WWW Document]. R Package version 0.99.2. Available from https://github.com/mhahsler/rBLAST.

[jkad253-B32] Hernández AF , Bennekou SH, Hart A, Mohimont L, Wolterink G. 2020. Mechanisms underlying disruptive effects of pesticides on the thyroid function. Curr Opin Toxicol. 19:34–41. doi:10.1016/j.cotox.2019.10.003.

[jkad253-B33] Hoopes G , Meng X, Hamilton JP, Achakkagari SR, de Alves Freitas Guesdes F, Bolger ME, Coombs JJ, Esselink D, Kaiser NR, Kodde L, et al 2022. Phased, chromosome-scale genome assemblies of tetraploid potato reveal a complex genome, transcriptome, and predicted proteome landscape underpinning genetic diversity. Mol Plant. 15(3):520–536. doi:10.1016/j.molp.2022.01.003. 35026436

[jkad253-B34] Howard NP , Peace C, Silverstein KAT, Poets A, Luby JJ, Vanderzande S, Durel C-E, Muranty H, Denancé C, van de Weg E. 2021. The use of shared haplotype length information for pedigree reconstruction in asexually propagated outbreeding crops, demonstrated for apple and sweet cherry. Hortic Res. 8(1):202. doi:10.1038/s41438-021-00637-5. 34465774 PMC8408172

[jkad253-B35] Jombart T , Ahmed I. 2011. adegenet 1.3-1: new tools for the analysis of genome-wide SNP data. Bioinformatics. 27(21):3070–3071. doi:10.1093/bioinformatics/btr521. 21926124 PMC3198581

[jkad253-B36] Joshi SG , Schaart JG, Groenwold R, Jacobsen E, Schouten HJ, Krens FA. 2011. Functional analysis and expression profiling of *HcrVf1* and *HcrVf2* for development of scab resistant cisgenic and intragenic apples. Plant Mol Biol. 75(6):579–591. doi:10.1007/s11103-011-9749-1. 21293908 PMC3057008

[jkad253-B37] Jung S , Lee T, Cheng C-H, Buble K, Zheng P, Yu J, Humann J, Ficklin SP, Gasic K, Scott K, et al 2019. 15 Years of GDR: new data and functionality in the Genome Database for Rosaceae. Nucleic Acids Res. 47(D1):D1137–D1145. doi:10.1093/nar/gky1000. 30357347 PMC6324069

[jkad253-B38] Khajuria YP , Kaul S, Wani AA, Dhar MK. 2018. Genetics of resistance in apple against *Venturia inaequalis* (Wint.) Cke. Tree Genet Genomes. 14(2):16. doi:10.1007/s11295-018-1226-4.

[jkad253-B39] Khan A , Carey SB, Gomez AS, Zhang H, Hargarten H, Hale H, Harkess A, Honaas L. 2022. A phased, chromosome-scale genome of ‘Honeycrisp’ apple (*Malus domestica*). GigaByte. 2022:1–15. doi:10.46471/gigabyte.69. PMC9693968 36824509

[jkad253-B40] Khan A , Korban SS. 2022. Breeding and genetics of disease resistance in temperate fruit trees: challenges and new opportunities. Theor Appl Genet. 135(11):3961–3985. doi:10.1007/s00122-022-04093-0. 35441862

[jkad253-B41] Köller W , Avila-Adame C, Olaya G, Zheng D. 2002. Resistance to strobilurin fungicides. ACS Symp Ser. 808:215–229. doi:10.1021/bk-2002-0808.ch014.

[jkad253-B42] Köller W , Wilcox WF. 2000. Interactive effects of dodine and the DMI fungicide fenarimol in the control of apple scab. Plant Dis. 84(8):863–870. doi:10.1094/PDIS.2000.84.8.863. 30832140

[jkad253-B43] Köller W , Wilcox WF, Barnard J, Jones AL, Braun PG. 1997. Detection and quantification of resistance of *Venturia inaequalis* populations to sterol demethylation inhibitors. Phytopathology. 87(2):184–190. doi:10.1094/PHYTO.1997.87.2.184. 18945140

[jkad253-B44] Korf I . 2004. Gene finding in novel genomes. BMC Bioinformatics. 5(1):59. doi:10.1186/1471-2105-5-59. 15144565 PMC421630

[jkad253-B45] Kurtz S , Phillippy A, Delcher AL, Smoot M, Shumway M, Antonescu C, Salzberg SL. 2004. Versatile and open software for comparing large genomes. Genome Biol. 5(2):1–9. doi:10.1186/gb-2004-5-2-r12. PMC395750 14759262

[jkad253-B46] Letunic I , Bork P. 2021. Interactive Tree Of Life (iTOL) v5: an online tool for phylogenetic tree display and annotation. Nucleic Acids Res. 49(W1):W293–W296. doi:10.1093/nar/gkab301. 33885785 PMC8265157

[jkad253-B47] Li H . 2021. New strategies to improve minimap2 alignment accuracy. Bioinformatics. 37(23):4572–4574. doi:10.1093/bioinformatics/btab705. 34623391 PMC8652018

[jkad253-B48] Li Z , Wang L, He J, Li X, Hou N, Guo J, Niu C, Li C, Liu S, Xu J, et al 2022. Chromosome-scale reference genome provides insights into the genetic origin and grafting-mediated stress tolerance of *Malus prunifolia*. Plant Biotechnol J. 20(6):1015–1017. doi:10.1111/pbi.13817. 35348283 PMC9129071

[jkad253-B49] MacHardy WE . 1996. Apple Scab: Biology, Epidemiology and Management. St Paul (MN): The American Phytopathological Society Press.

[jkad253-B50] Malnoy M , Xu M, Borejsza-wysocka E, Korban SS, Aldwinckle HS. 2008. Two receptor-like genes, *Vfa1* and *Vfa2*, confer resistance to the fungal pathogen *Venturia inaequalis* inciting apple scab disease. Mol Plant Microbe Interact. 21(4):448–458. doi:10.1094/MPMI-21-4-0448. 18321190

[jkad253-B51] Manni M , Berkeley MR, Seppey M, Simão FA, Zdobnov EM. 2021. BUSCO update: novel and streamlined workflows along with broader and deeper phylogenetic coverage for scoring of eukaryotic, prokaryotic, and viral genomes. Mol Biol Evol. 38(10):4647–4654. doi:10.1093/molbev/msab199. 34320186 PMC8476166

[jkad253-B52] Marçais G , Kingsford C. 2011. A fast, lock-free approach for efficient parallel counting of occurrences of k-mers. Bioinformatics. 27(6):764–770. doi:10.1093/bioinformatics/btr011. 21217122 PMC3051319

[jkad253-B53] Minio A , Cochetel N, Vondras AM, Massonnet M, Cantu D. 2022. Assembly of complete diploid-phased chromosomes from draft genome sequences. G3 (Bethesda). 12(8):jkac143. doi:10.1093/g3journal/jkac143. 35686922 PMC9339290

[jkad253-B54] Mulder N , Apweiler R. 2007. InterPro and InterProScan: tools for protein sequence classification and comparison. Comp Genomics. 396:59–70. doi:10.1385/1-59745-515-6:59. 18025686

[jkad253-B55] Nattestad M , Schatz MC. 2016. Assemblytics: a web analytics tool for the detection of variants from an assembly. Bioinformatics. 32(19):3021–3023. doi:10.1093/bioinformatics/btw369. 27318204 PMC6191160

[jkad253-B56] Ou S , Su W, Liao Y, Chougule K, Agda JRA, Hellinga AJ, Lugo CSB, Elliott TA, Ware D, Peterson T, et al 2019. Benchmarking transposable element annotation methods for creation of a streamlined, comprehensive pipeline. Genome Biol. 20(1):275. doi:10.1186/s13059-019-1905-y. 31843001 PMC6913007

[jkad253-B57] Padmarasu S , Sargent DJ, Patocchi A, Troggio M, Baldi P, Linsmith G, Poles L, Jänsch M, Kellerhals M, Tartarini S, et al 2018. Identification of a leucine-rich repeat receptor-like serine/threonine-protein kinase as a candidate gene for *Rvi12 (Vb*)-based apple scab resistance. Mol Breed. 38(6):73. doi:10.1007/s11032-018-0825-y.

[jkad253-B58] Papp D , Singh J, Gadoury DM, Khan A. 2019. New North American isolates of *Venturia inaequalis* can overcome apple scab resistance of *Malus floribunda* 821. Plant Dis. 104(3):649–655. doi:10.1094/pdis-10-19-2082-re. 31961770

[jkad253-B59] Parisi L , Lespinasse Y. 1996. Pathogenicity of *Venturia inaequalis* strains of race 6 on apple clones (*Malus* sp.). Plant Dis. 80(10):1179–1183. doi:10.1094/PD-80-1179.

[jkad253-B60] Parisi L , Lespinasse Y, Guillaumes J, Krüger J. 1993. A new race of *Venturia inaequalis* virulent to apples with resistance due to the *Vf* gene. Phytopathology. 83(5):533–537. doi:10.1094/Phyto-83-533.

[jkad253-B61] Patocchi A , Vinatzer BA, Gianfranceschi L, Tartarini S, Zhang HB, Sansavini S, Gessler C. 1999. Construction of a 550 kb BAC contig spanning the genomic region containing the apple scab resistance gene *Vf*. Mol Gen Genet. 262(4–5):884–891. doi:10.1007/s004380051154. 10628874

[jkad253-B62] Patocchi A , Wehrli A, Dubuis P-H, Auwerkerken A, Leida C, Cipriani G, Passey T, Staples M, Didelot F, Philion V, et al 2020. Ten years of VINQUEST: first insight for breeding new apple cultivars with durable apple scab resistance. Plant Dis. 104(8):2074–2081. doi:10.1094/PDIS-11-19-2473-SR. 32525450

[jkad253-B63] Paysan-Lafosse T , Blum M, Chuguransky S, Grego T, Pinto BL, Salazar GA, Bileschi ML, Bork P, Bridge A, Colwell L, et al 2023. InterPro in 2022. Nucleic Acids Res. 51(D1):D418–D427. doi:10.1093/nar/gkac993. 36350672 PMC9825450

[jkad253-B64] Perazzolli M , Malacarne G, Baldo A, Righetti L, Bailey A, Fontana P, Velasco R, Malnoy M. 2014. Characterization of resistance gene analogues (RGAs) in apple (*Malus* x *domestica* Borkh.) and their evolutionary history of the Rosaceae family. PLoS One. 9(2):e83844. doi:10.1371/journal.pone.0083844. 24505246 PMC3914791

[jkad253-B65] Pikunova A , Madduri M, Sedov E, Noordijk Y, Peil A, Troggio M, Bus VGM, Visser RGF, van de Weg E. 2014. ‘Schmidt's Antonovka’ is identical to ‘Common Antonovka’, an apple cultivar widely used in Russia in breeding for biotic and abiotic stresses. Tree Genet Genomes. 10(2):261–271. doi:10.1007/s11295-013-0679-8.

[jkad253-B66] Raghavan V . 2021. seqvisr (v0.2.5). Zenodo. 10.5281/zenodo.6583981.

[jkad253-B67] Ranallo-Benavidez TR , Jaron KS, Schatz MC. 2020. GenomeScope 2.0 and Smudgeplot for reference-free profiling of polyploid genomes. Nat Commun. 11(1):1432. doi:10.1038/s41467-020-14998-3. 32188846 PMC7080791

[jkad253-B68] Rhie A , Walenz BP, Koren S, Phillippy AM. 2020. Merqury: reference-free quality, completeness, and phasing assessment for genome assemblies. Genome Biol. 21(1):1–27. doi:10.1186/s13059-020-02134-9. PMC7488777 32928274

[jkad253-B69] Schouten HJ , Brinkhuis J, van der Burgh A, Schaart JG, Groenwold R, Broggini GAL, Gessler C. 2014. Cloning and functional characterization of the *Rvi15 (Vr2*) gene for apple scab resistance. Tree Genet Genomes. 10(2):251–260. doi:10.1007/s11295-013-0678-9.

[jkad253-B70] Schultz D , De Coster W, Nicholls M. 2018. conchoecia/pauvre: DOI initialize (0.1.86). Zenodo. 10.5281/zenodo.1184706.

[jkad253-B71] Shirane N , Takenaka H, Ueda K, Hashimoto Y, Katoh K, Ishii H. 1996. Sterol analysis of DMI-resistant and -sensitive strains of *Venturia inaequalis*. Phytochemistry. 41(5):1301–1308. doi:10.1016/0031-9422(95)00787-3.

[jkad253-B72] Silfverberg-Dilworth E , Besse S, Paris R, Belfanti E, Tartarini S, Sansavini S, Patocchi A, Gessler C. 2005. Identification of functional apple scab resistance gene promoters. Theor Appl Genet. 110(6):1119–1126. doi:10.1007/s00122-005-1940-9. 15726316

[jkad253-B73] Singh J , Sun M, Cannon SB, Wu J, Khan A. 2021. An accumulation of genetic variation and selection across the disease-related genes during apple domestication. Tree Genet Genomes. 17(3):1–11. doi:10.1007/s11295-021-01510-1.

[jkad253-B74] Srivastava AK , Singh BP, Kaushik SK, Bhardwaj V, Tiwari JK, Sharma S. 2018. Identification of late blight resistance gene homologues in wild Solanum species. Proc Natl Acad Sci India Sect B Biol Sci. 88(2):789–796. doi:10.1007/s40011-016-0811-2.

[jkad253-B75] Stanke M , Keller O, Gunduz I, Hayes A, Waack S, Morgenstern B. 2006. AUGUSTUS: ab initio prediction of alternative transcripts. Nucleic Acids Res. 34(Web Server):W435–W439. doi:10.1093/nar/gkl200. 16845043 PMC1538822

[jkad253-B76] Sun X , Jiao C, Schwaninger H, Chao CT, Ma Y, Duan N, Khan A, Ban S, Xu K, Cheng L, et al 2020. Phased diploid genome assemblies and pan-genomes provide insights into the genetic history of apple domestication. Nat Genet. 52(12):1423–1432. doi:10.1038/s41588-020-00723-9. 33139952 PMC7728601

[jkad253-B77] Tamura K , Stecher G, Kumar S. 2021. MEGA11: molecular evolutionary genetics analysis version 11. Mol Biol Evol. 38(7):3022–3027. doi:10.1093/molbev/msab120. 33892491 PMC8233496

[jkad253-B78] Tartarini S , Gianfranceschi L, Sansavini S, Gessler C. 1999. Development of reliable PCR markers for the selection of the *Vf* gene conferring scab resistance in apple. Plant Breed. 118(2):183–186. doi:10.1046/j.1439-0523.1999.118002183.x.

[jkad253-B79] Tatusova T , DiCuccio M, Badretdin A, Chetvernin V, Nawrocki EP, Zaslavsky L, Lomsadze A, Pruitt KD, Borodovsky M, Ostell J. 2016. NCBI prokaryotic genome annotation pipeline. Nucleic Acids Res. 44(14):6614–6624. doi:10.1093/nar/gkw569. 27342282 PMC5001611

[jkad253-B80] Turechek WW . 2004. Apple diseases and their management. In: Naqvi SAMH, editor. Diseases of Fruits and Vegetables Volume I: Diagnosis and Management. Dordrecht (The Netherlands): Springer. p. 1–108.

[jkad253-B81] Turechek WW , Köller W. 2004. Managing resistance of *Venturia inaequalis* to the strobilurin fungicides. Plant Health Prog. 5:1–8. doi:10.1094/PHP-2004-0908-01-RS.

[jkad253-B82] Uliano-Silva M , Ferreira GR, Krasheninnikova J, Darwin Tree of Life Consortium, Formenti G, Abueg L, Torrance J, Myers EW, Durbin R, et al 2022. MitoHiFi: a python pipeline for mitochondrial genome assembly from PacBio high fidelity reads. bioRxiv 521667. 10.1101/2022.12.23.521667, preprint: not peer reviewed.

[jkad253-B83] Vinatzer BA , Patocchi A, Gianfranceschi L, Tartarini S, Zhang HB, Gessler C, Sansavini S. 2001. Apple contains receptor-like genes homologous to the *Cladosporium fulvum* resistance gene family of tomato with a cluster of genes cosegregating with *Vf* apple scab resistance. Mol Plant Microbe Interact. 14(4):508–515. doi:10.1094/MPMI.2001.14.4.508. 11310738

[jkad253-B84] Vinatzer BA , Patocchi A, Tartarini S, Gianfranceschi L, Sansavini S, Gessler C. 2004. Isolation of two microsatellite markers from BAC clones of the *Vf* scab resistance region and molecular characterization of scab-resistant accessions in *Malus* germplasm. Plant Breed. 123(4):321–326. doi:10.1111/j.1439-0523.2004.00973.x.

[jkad253-B85] Wilner J . 1960. Relative and absolute electrolytic conductance tests for frost hardiness of apple varieties. Can J Plant Sci. 40(4):630–637. doi:10.4141/cjps60-093.

[jkad253-B86] Xu M , Korban SS. 2002. A cluster of four receptor-like genes resides in the *Vf* locus that confers resistance to apple scab disease. Genetics. 162(4):1995–2006. doi:10.1093/genetics/162.4.1995. 12524365 PMC1462389

[jkad253-B87] Xu M , Korban SS. 2004. Somatic variation plays a key role in the evolution of the *Vf* gene family residing in the *Vf* locus that confers resistance to apple scab disease. Mol Phylogenet Evol. 32(1):57–65. doi:10.1016/j.ympev.2003.12.004. 15186797

[jkad253-B88] Zaller JG , Brühl CA. 2019. Editorial: non-target effects of pesticides on organisms inhabiting agroecosystems. Front Environ Sci. 7:75. doi:10.3389/fenvs.2019.00075.

[jkad253-B89] Zhang L , Hu J, Han X, Li J, Gao Y, Richards CM, Zhang C, Tian Y, Liu G, Gul H, et al 2019. A high-quality apple genome assembly reveals the association of a retrotransposon and red fruit colour. Nat Commun. 10(1):1494. doi:10.1038/s41467-019-09518-x. 30940818 PMC6445120

