# Supplementary material for: Chromosome-level phased genome assembly of “Antonovka” identified candidate apple scab-resistance genes highly homologous to HcrVf2 and HcrVf1 on linkage group 1
Source: G3 (Bethesda). 2023 Nov 4;14(1):jkad253. doi: 10.1093/g3journal/jkad253 (PMC10755186; doi:10.1093/g3journal/jkad253)
Supplement: jkad253_Supplementary_Data [file jkad253_supplementary_data.zip › Supplementary_figures_and_tables_G3-2023-404467.docx]

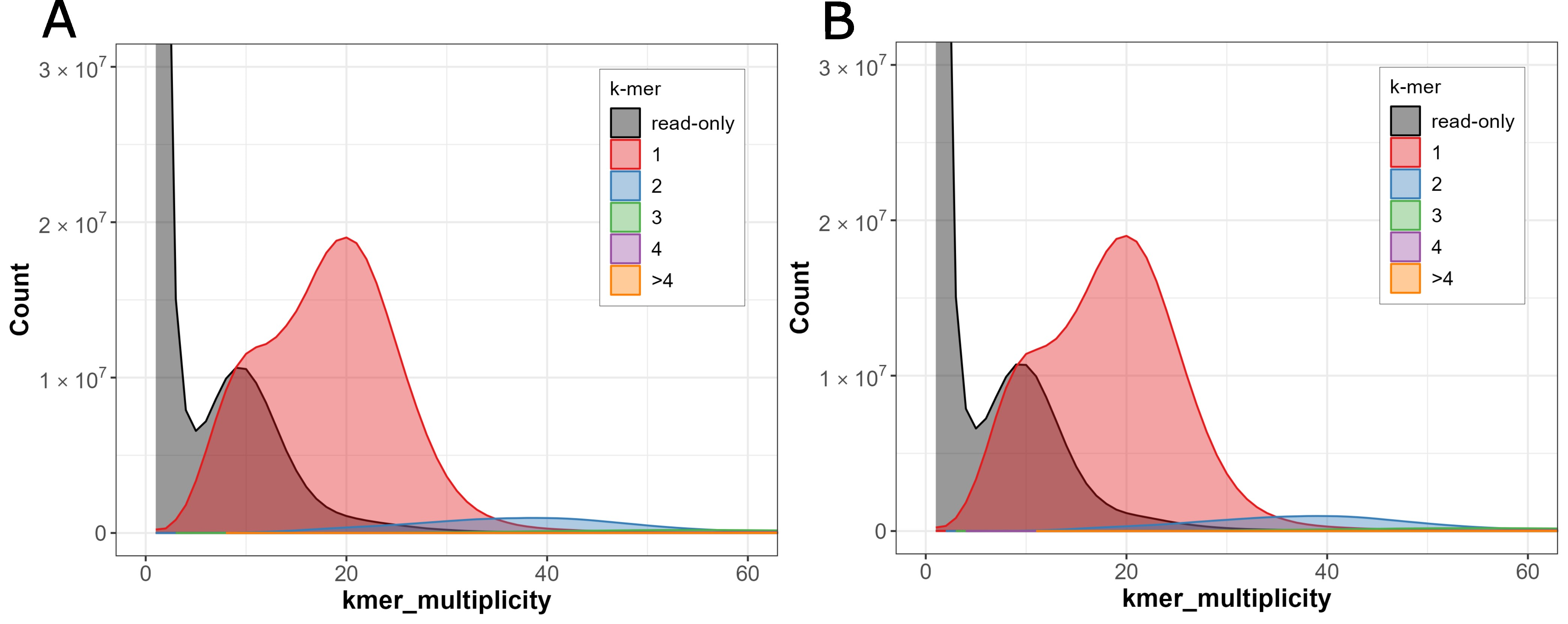


**Figure S1:** Histogram of *k*-mer multiplicity of sequencing reads of apple ‘Antonovka’ 172670-B. (A) Haplome A and (B) Haplome B of ‘Antonovka’ 172670-B genome assemblies. *k*-mer multiplicity (x-axis) is plotted against *k*-mer counts (y-axis) to estimate the heterozygosity, copy numbers, sequencing depth, and completeness of a genome using Merqury v1.3 (Rhie et al., 2020). Colors in the plot represent the number of times each *k*-mer is found in the genome assembly.


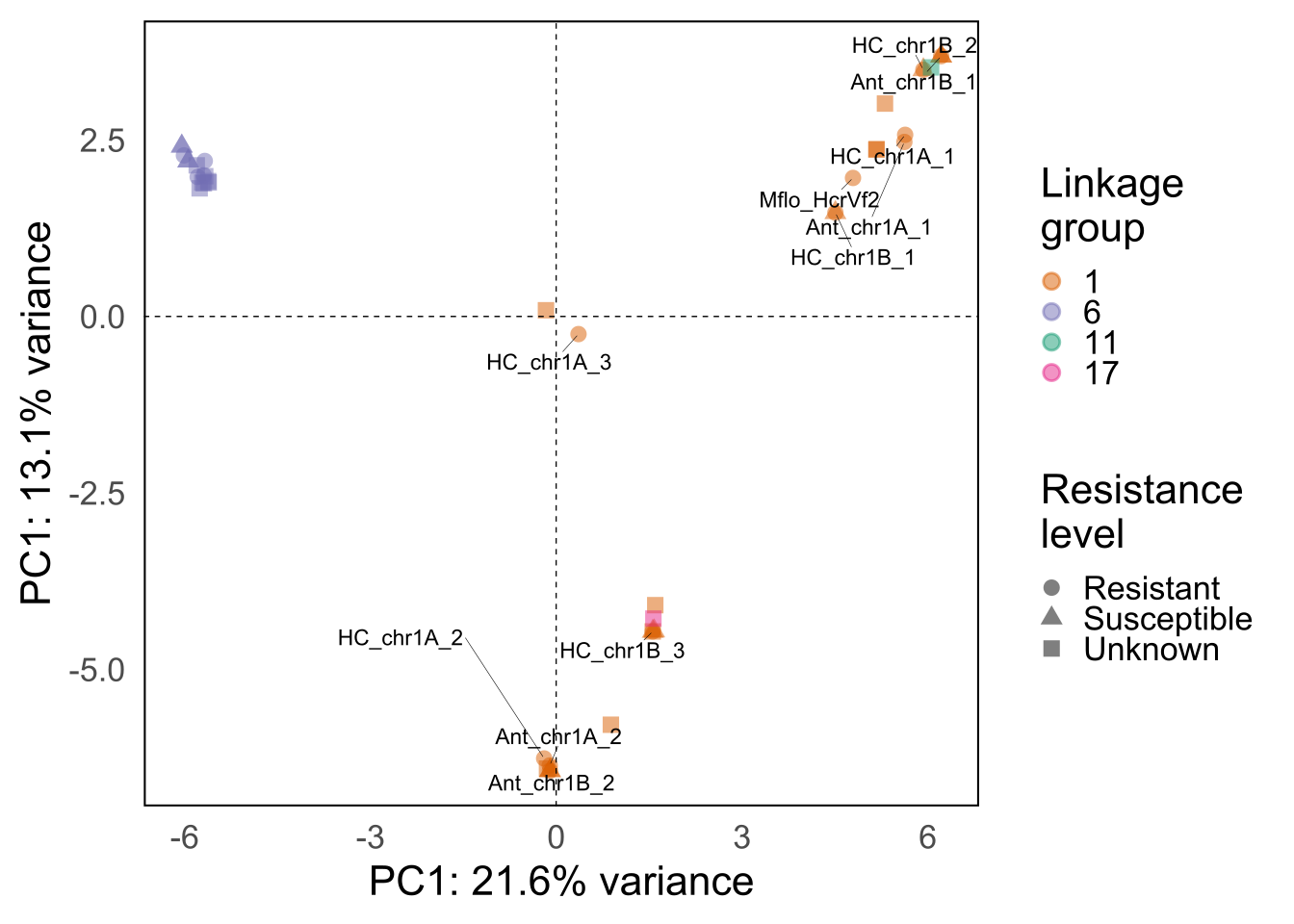


**Figure S2:** Principal component analysis (PCA) of sequence variation among *HcrVf2* from linkage group 1 of *M. floribunda* 821 homologs in nine apple genomes. Plot shows the first and the second principal component (PC1 and PC2, respectively) and variance explained by each PC. Sequence names indicate accession/cultivar name, followed by a chromosome number and haplotype. Resistance level indicates resistance to apple scab of the analyzed accession in which a specific homologous sequence has been identified. The first part of the identification name represents accession/cultivar name (HC: ‘Honeycrisp’; Ant: ‘Antonovka’ 172670-B; Mflo: *M. floribunda* 821), followed by a chromosome number, haplome (if available), and the ranking based on the blast score from the same genotype.


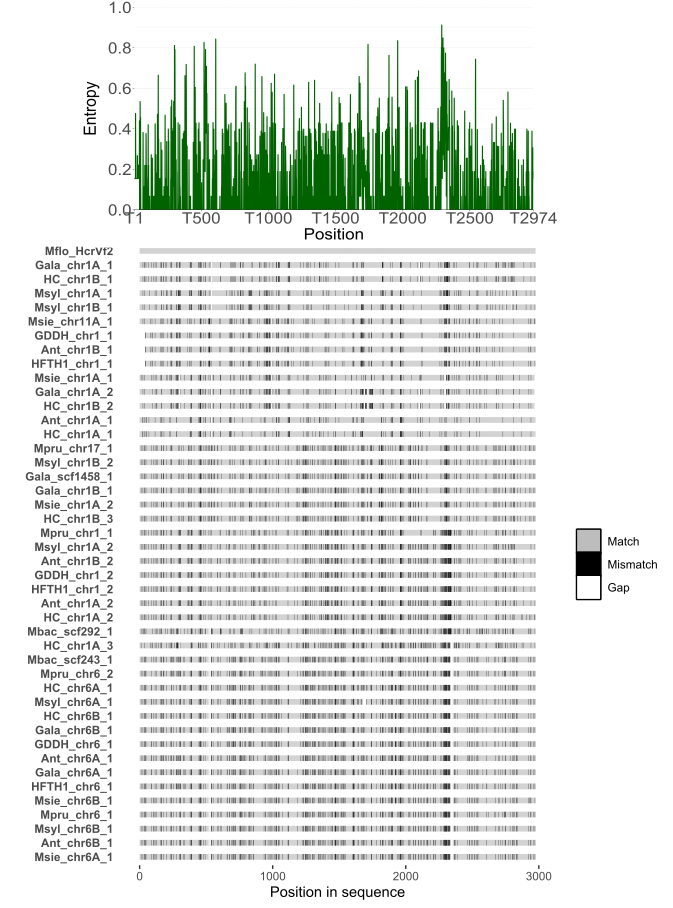


**Figure S3:** Alignment analysis of the sequences identified in nine apple genomes that are homologous to *HcrVf2* from linkage group 1 of *M. floribunda* 821. A) Sequence entropy by position (x axis) from the nucleotide 1 (T1) to 2974 (T2974) of *HcrVf2* across all the identified homologs. B) Sequence variation along the sequence alignment from nucleotide 1 to 2974 of *HcrVf2* (x axis-Position in sequence). Sequence names indicate accession/cultivar name, followed by a chromosome number and haplotype. Gray, black, and white color indicate nucleotides that match, do not match, and are not present, respectively, in a homolog compared to *HcrVf2* at a specific position. The first part of the identification name represents accession/cultivar name (HC: ‘Honeycrisp’; Ant: ‘Antonovka’ 172670-B; Mflo: *M. floribunda* 821; Mbac; *M. baccata*; Mpru: *M. prunifolia*; Msyl: *M. sylvestris*; Msie: *M. sieversii*; HFTH1: anther-derived homozygous genotype HFTH1; GDDH13: Doubled-haploid derivative of ‘Golden Delicious’), followed by a chromosome number, haplome (if available), and the ranking based on the blast score from the same genotype.


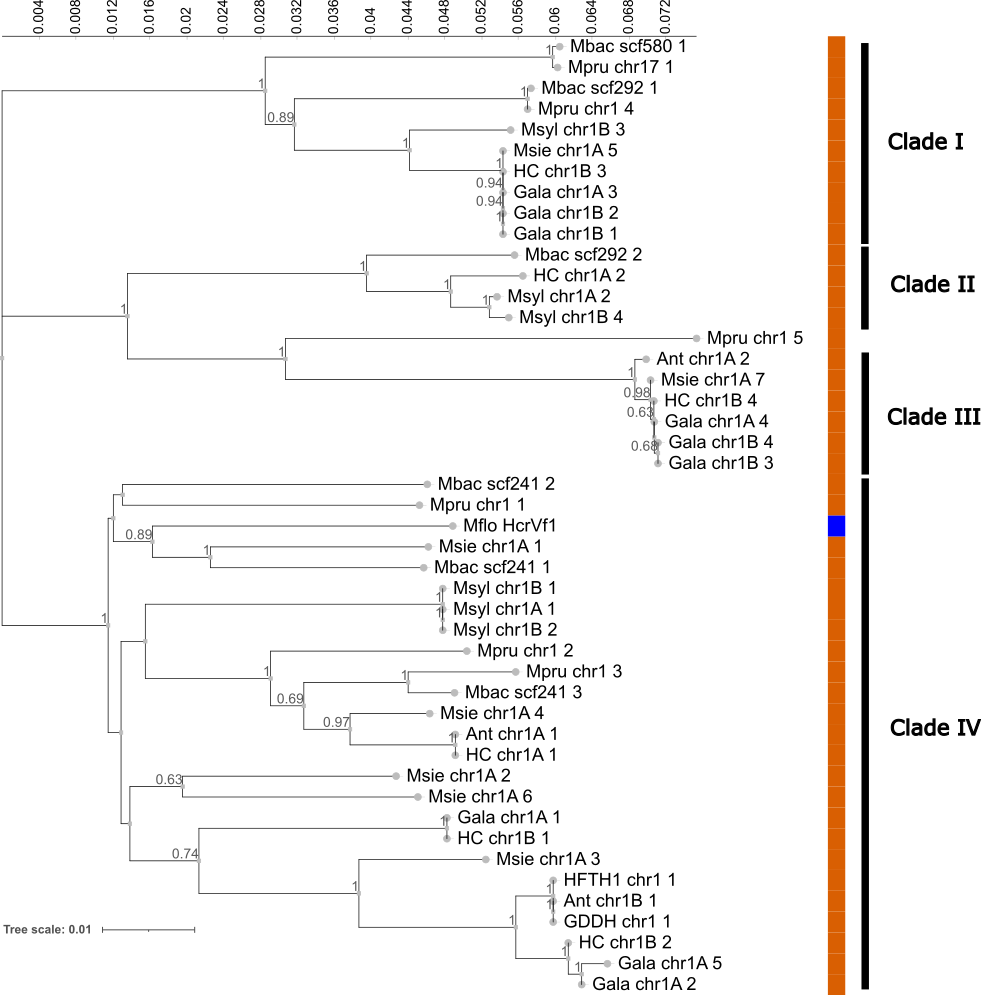


**Figure S4:** Phylogenetic tree of *HcrVf1* homologs from 12 genome assemblies of *Malus* *domestica* and wild *Malus* genotypes generated by the neighbor-joining phylogenetic analysis in MEGA11 (Tamura et al., 2021). The 43 homologs of *HcrVf1* from *Malus* form four major clades, supported by bootstrap numbers. All members in clades I, II, III, and IV are located on chromosome 1 or are placed to linkage groups 17, or a scaffold (orange). Within clade IV, *HcrVf*1 from *Malus floribunda* 821 can be found (blue). The first part of the sequence identification name represents accession/cultivar name, followed by a chromosome number, haplotype (if available), and the ranking based on the blast score from the same genotype. Numbers on nodes are bootstrap values, and values <0.50 are not shown. The first part of the identification name represents accession/cultivar name (HC: ‘Honeycrisp’; Ant: ‘Antonovka’ 172670-B; Mflo: *M. floribunda* 821; Mbac; *M. baccata*; Mpru: *M. prunifolia*; Msyl: *M. sylvestris*; Msie: *M. sieversii*; HFTH1: anther-derived homozygous genotype HFTH1; GDDH13: Doubled-haploid derivative of ‘Golden Delicious’), followed by a chromosome number, haplome (if available), and the ranking based on the blast score from the same genotype.


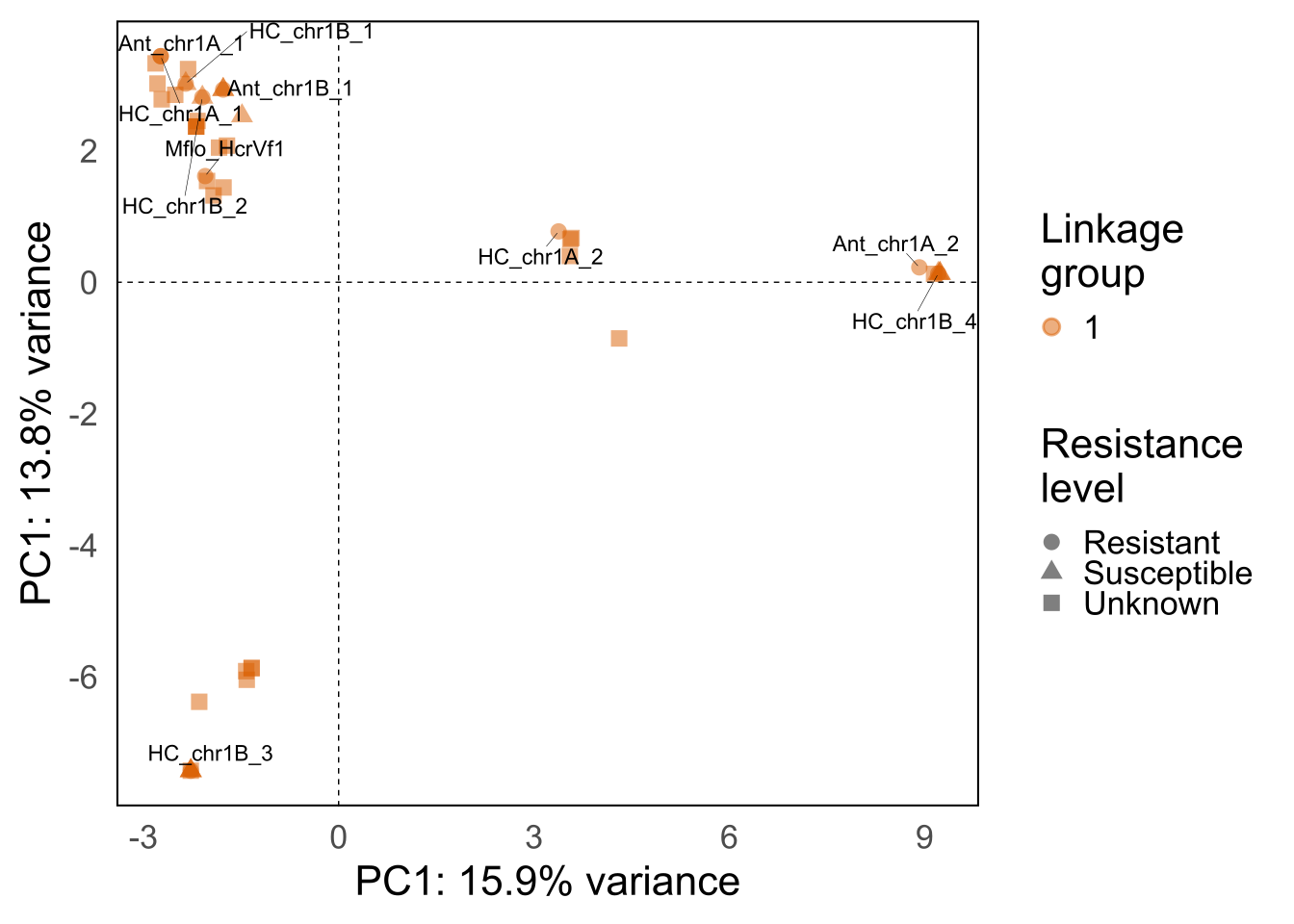


**Figure S5:** Principal component analysis (PCA) of sequence variation among HcrVf1 from linkage group 1 of M. floribunda 821 homologs in nine apple genomes. Plot shows the first and the second principal component (PC1 and PC2, respectively) and variance explained by each PC. Sequence names indicate accession/cultivar name, followed by a chromosome number and haplotype. Resistance level indicates resistance to apple scab of the analyzed accession in which a specific homologous sequence has been identified. The first part of the identification name represents accession/cultivar name (HC: ‘Honeycrisp’; Ant: ‘Antonovka’ 172670-B; Mflo: M. floribunda 821), followed by a chromosome number, haplome (if available), and the ranking based on the blast score from the same genotype.


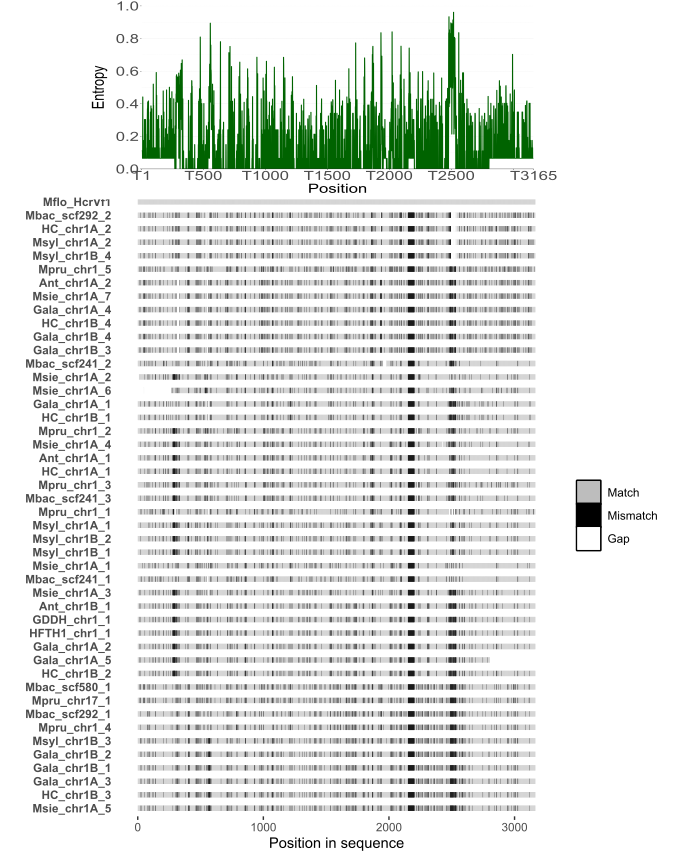


**Figure S6:** Alignment analysis of the sequences identified in nine apple genomes that are homologous to *HcrVf1* from linkage group 1 of *M. floribunda* 821. A) Sequence entropy by position (x axis) from the nucleotide 1 (T1) to 3165 (T3165) of *HcrVf1* across all the identified homologs. B) Sequence variation along the sequence alignment from nucleotide 1 to 3165 of *HcrVf1* (x axis-Position in sequence). Sequence names indicate accession/cultivar name, followed by a chromosome number and haplotype. Gray, black, and white color indicate nucleotides that match, do not match, and are not present, respectively, in a homolog compared to *HcrVf1* at a specific position. The first part of the identification name represents accession/cultivar name (HC: ‘Honeycrisp’; Ant: ‘Antonovka’ 172670-B; Mflo: *M. floribunda* 821; Mbac; *M. baccata*; Mpru: *M. prunifolia*; Msyl: *M. sylvestris*; Msie: *M. sieversii*; HFTH1: anther-derived homozygous genotype HFTH1; GDDH13: Doubled-haploid derivative of ‘Golden Delicious’), followed by a chromosome number, haplome (if available), and the ranking based on the blast score from the same genotype.


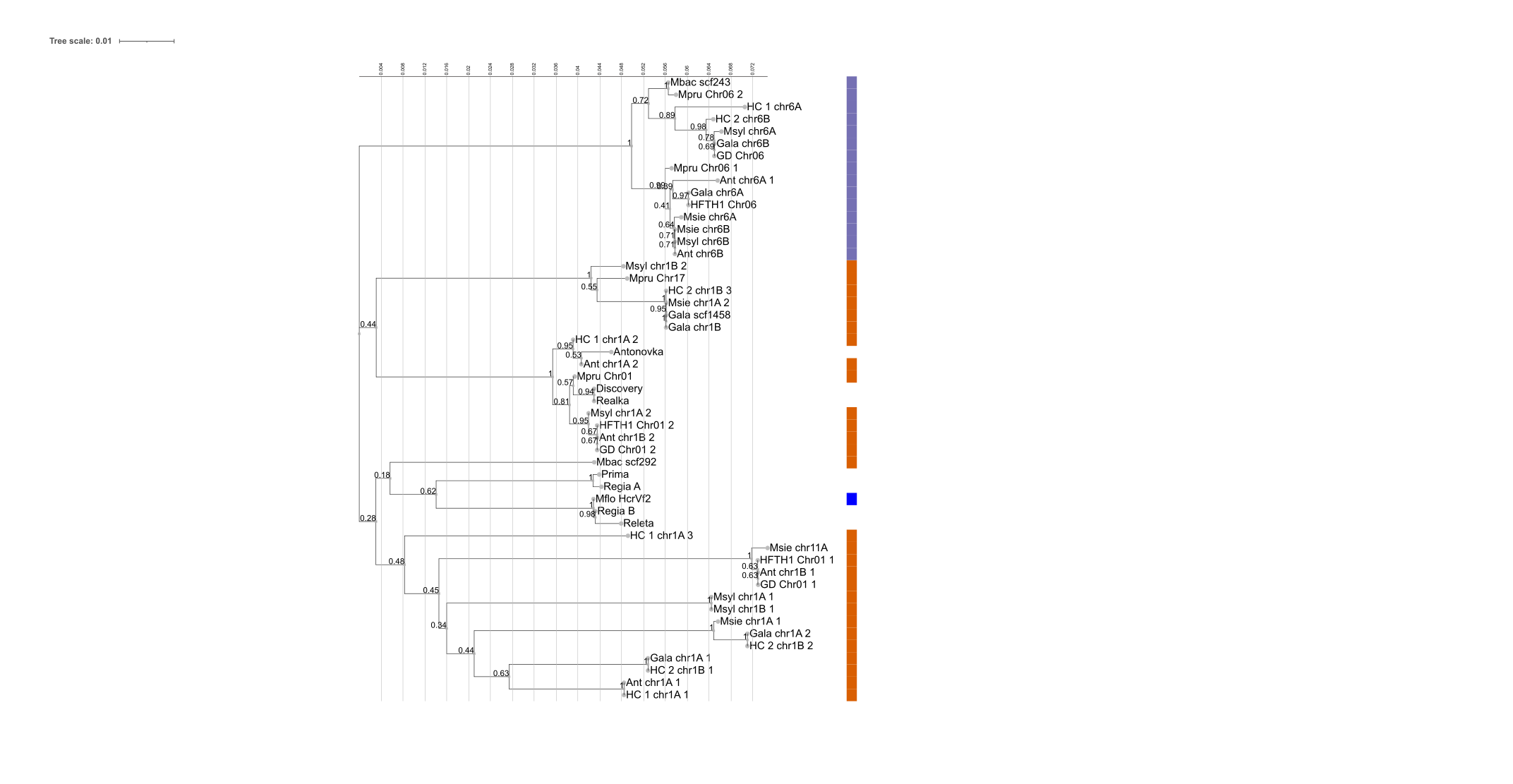


**Figure S7:** Phylogenetic tree of HcrVf2 PCR products from nine genome assemblies of Malus domestica and wild Malus genotypes based on the study of Budichevskaia et al. (2009) of about 700 and 800 bp generated by neighbor-joining phylogenetic analysis in MEGA11 (Tamura et al., 2021). The 43 homologs of HcrVf2 from Malus identified in our study, and the seven homologs from Budichevskaia et al. (2009) form only a single major clade (therefore no cladding is indicated in the figure), supported by bootstrap numbers. Members located on chromosome 1, 11, 17, or a scaffold (orange), chromosome 6 or on an unplaced scaffold (purple), and HcrVf2 from Malus floribunda 821 are indicated (blue). The first part of the sequence identification name represents accession/cultivar name, followed by a chromosome number, haplotype (if available), and the ranking based on the blast score from the same genotype. Numbers on nodes are bootstrap values, and values <0.50 are not shown. The first part of the identification name represents accession/cultivar name (HC: ‘Honeycrisp’; Ant: ‘Antonovka’ 172670-B; Mflo: M. floribunda 821; Mbac; M. baccata; Mpru: M. prunifolia; Msyl: M. sylvestris; Msie: M. sieversii; HFTH1: anther-derived homozygous genotype HFTH1; GDDH13: Doubled-haploid derivative of ‘Golden Delicious’), followed by a chromosome number, haplome (if available), and the ranking based on the blast score from the same genotype.


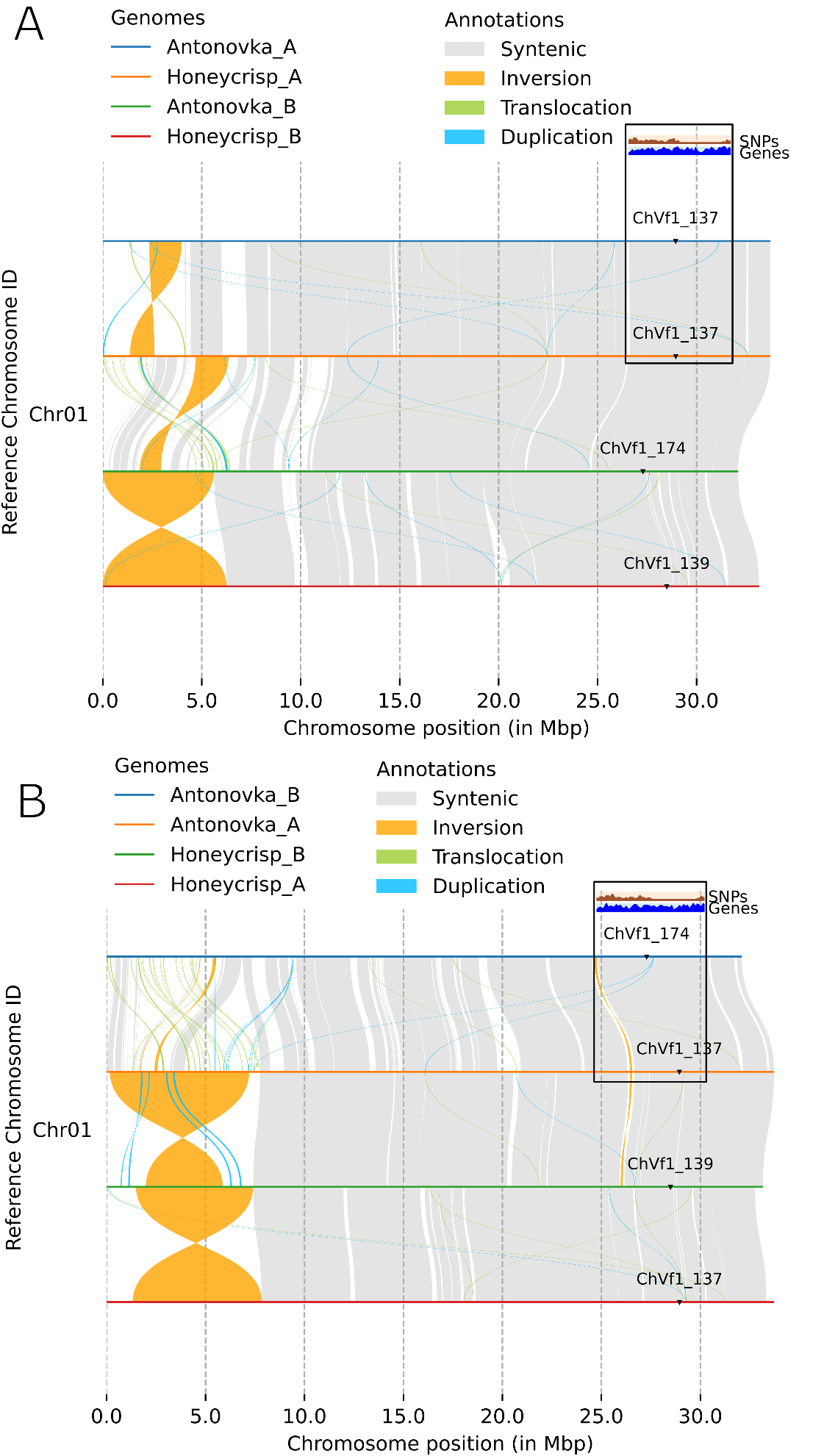


**Figure S8:** Synteny plots of the linkage group 1 haplomes A and B from ‘Antonovka’ 172670-B and ‘Honeycrisp’, indicating the position and size of the Ch-Vf1 scab resistance marker, and SNP and gene density along the up- and downstream flanking regions of Ch-Vf1. Panels A) and B) indicate all combinations of pairwise synteny comparisons between the four haplomes.


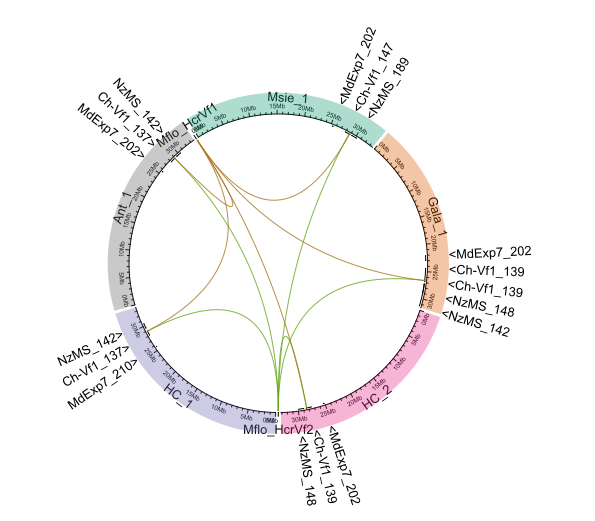


**Figure S9:** Schematic illustration of the location of HcrVf homologs on linkage group 1 of Malus floribunda 821 as a circoplot showing physical positions of the most related homologs to HcrVf2 (green lines) and HcrVf1 (brown lines) in haplome A of ‘Honeycrisp’ (HC_1) and ‘Antonovka’ 172670-B (Ant_1), Gala (Gala_1), haplome B of ‘Honeycrisp’ (HC_2), and Malus sieversii (Msie_1) relative to the location of the Ch-Vf1, MdExp7, and NzMS microsatellite markers.


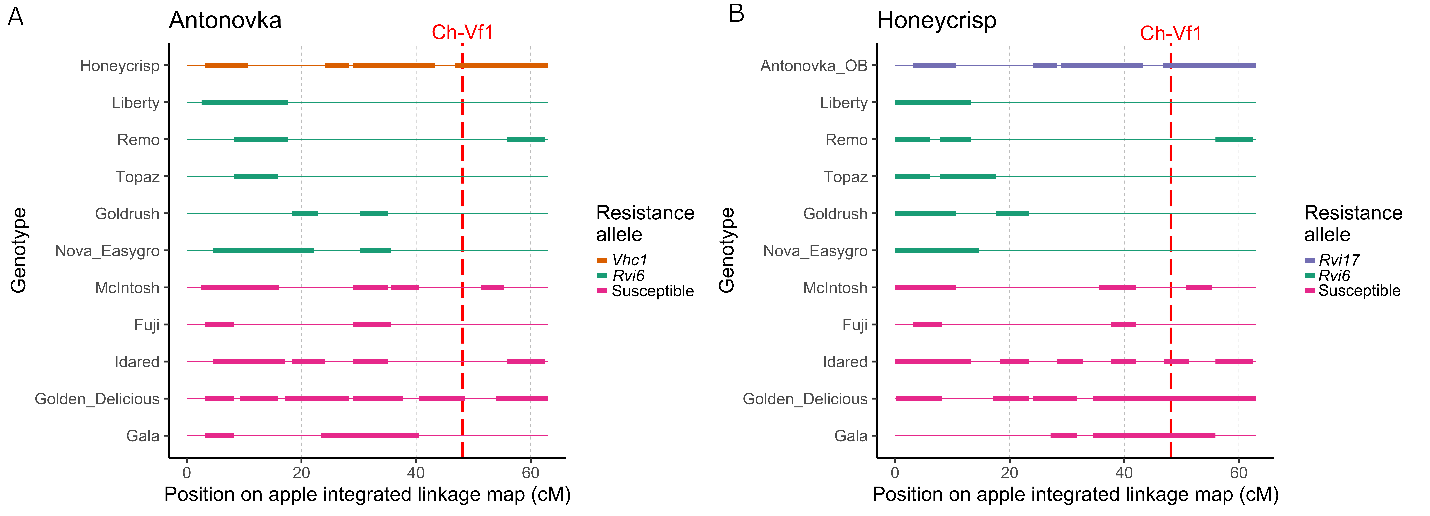
**Figure S10:** Extended shared haplotypes of the SNP marker data across the linkage group (LG) 1 from *Rvi17*-harboring ‘Antonovka’ (Common Antonovka) and B) LG 1 from *Vhc1*-harboring ‘Honeycrisp’, with different accessions known to carry different resistances, *i.e.,* ‘Honeycrisp’ and ‘Antonovka’, respectively, as well as *Rvi6* from *M. floribunda* 821, or those found to be susceptible to the majority of *Venturia inaequalis* races. Thin and bold lines indicate SNP allelic regions that are not shared/-identical and shared/-identical, respectively, with the SNPs in ‘Antonovka’ and ‘Honeycrisp’. The red dashed vertical lines indicate the approximate location of the Ch-Vf1 marker on the genetic map.


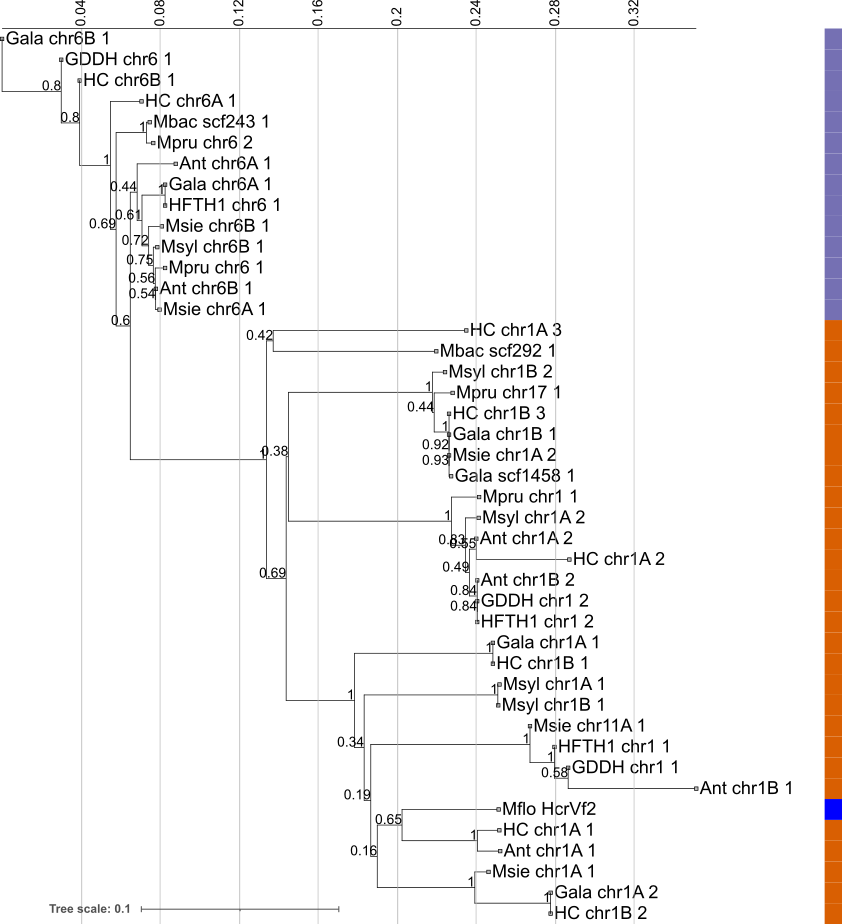


**Figure S11:** Phylogenetic tree of HcrVf2 homologs from different genotypes of Malus based on their amino acid sequences. The 43 homologs of HcrVf2 from Malus form a single clade, supported by bootstrap numbers. All the members in the clade are located on linkage group (LG) 1 or are exceptionally placed to LG 11, 17, or a scaffold (orange), LG 6 or on an unplaced scaffold (purple), or are the HcrVf1 from Malus floribunda 821(blue). The first part of the sequence identification name represents accession/cultivar name, followed by a chromosome number, haplotype (if available), and the ranking based on the blast score from the same genotype. Numbers on nodes are bootstrap values, and values <0.50 are not shown. Malus_sylvestris_chr6a_1 has been removed as an outlier. The first part of the identification name represents accession/cultivar name (HC: ‘Honeycrisp’; Ant: ‘Antonovka’ 172670-B; Mflo: M. floribunda 821; Mbac; M. baccata; Mpru: M. prunifolia; Msyl: M. sylvestris; Msie: M. sieversii; HFTH1: anther-derived homozygous genotype HFTH1; GDDH13: Doubled-haploid derivative of ‘Golden Delicious’), followed by a chromosome number, haplome (if available), and the ranking based on the blast score from the same genotype.


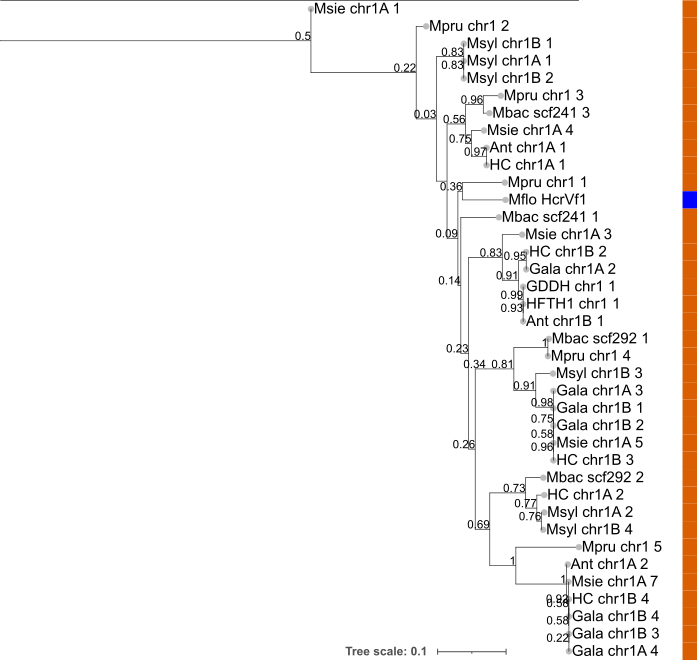


**Figure S12:** Phylogenetic tree of HcrVf1 homologs from different genotypes of Malus based on their amino acid sequences. The 46 homologs of HcrVf1 from Malus form a single major clade, supported by bootstrap numbers. All the members in the clade are located on linkage group (LG) 1 or a scaffold (orange color), or are theHcrVf1 from Malus floribunda 821 (blue color). The first part of the sequence identification name represents accession/cultivar name, followed by a chromosome number, haplotype (if available), and the ranking based on the blast score from the same genotype. Numbers on nodes are bootstrap values, and values <0.50 are not shown. Several outliers were removed. The first part of the identification name represents accession/cultivar name (HC: ‘Honeycrisp’; Ant: ‘Antonovka’ 172670-B; Mflo: M. floribunda 821; Mbac; M. baccata; Mpru: M. prunifolia; Msyl: M. sylvestris; Msie: M. sieversii; HFTH1: anther-derived homozygous genotype HFTH1; GDDH13: Doubled-haploid derivative of ‘Golden Delicious’), followed by a chromosome number, haplome (if available), and the ranking based on the blast score from the same genotype.

***
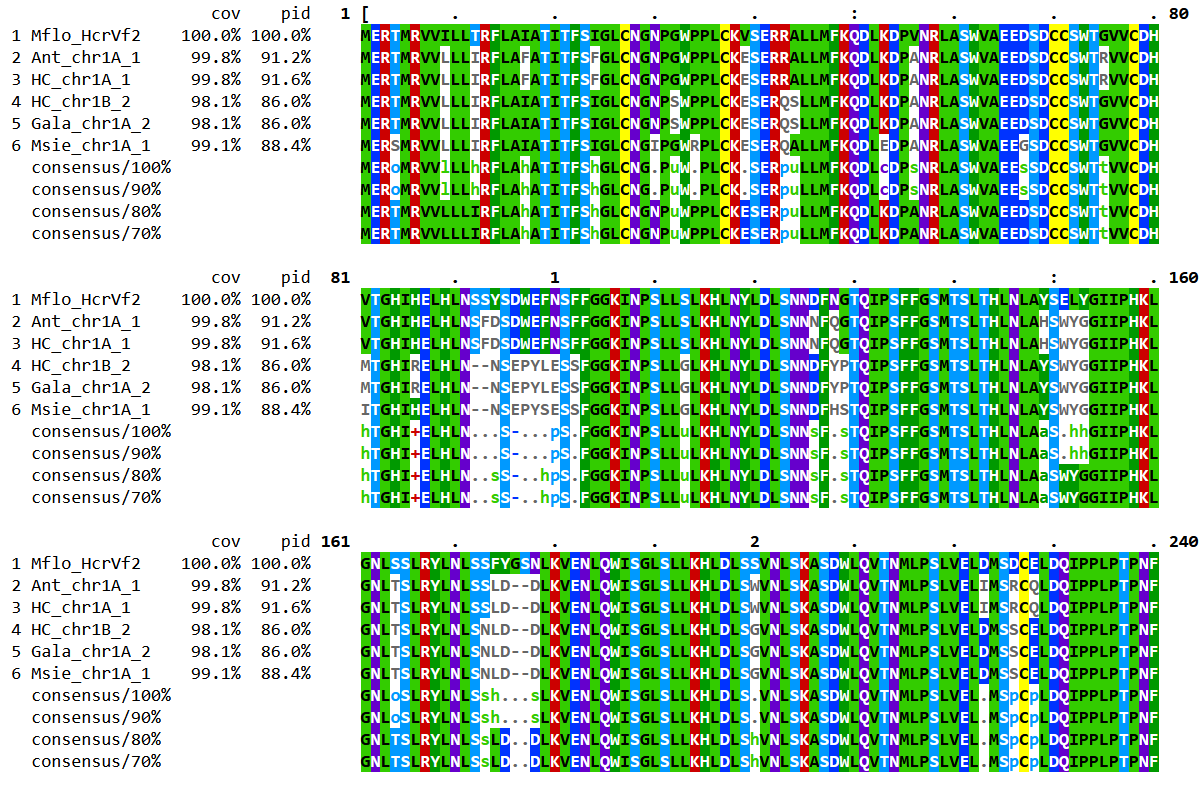
***

***
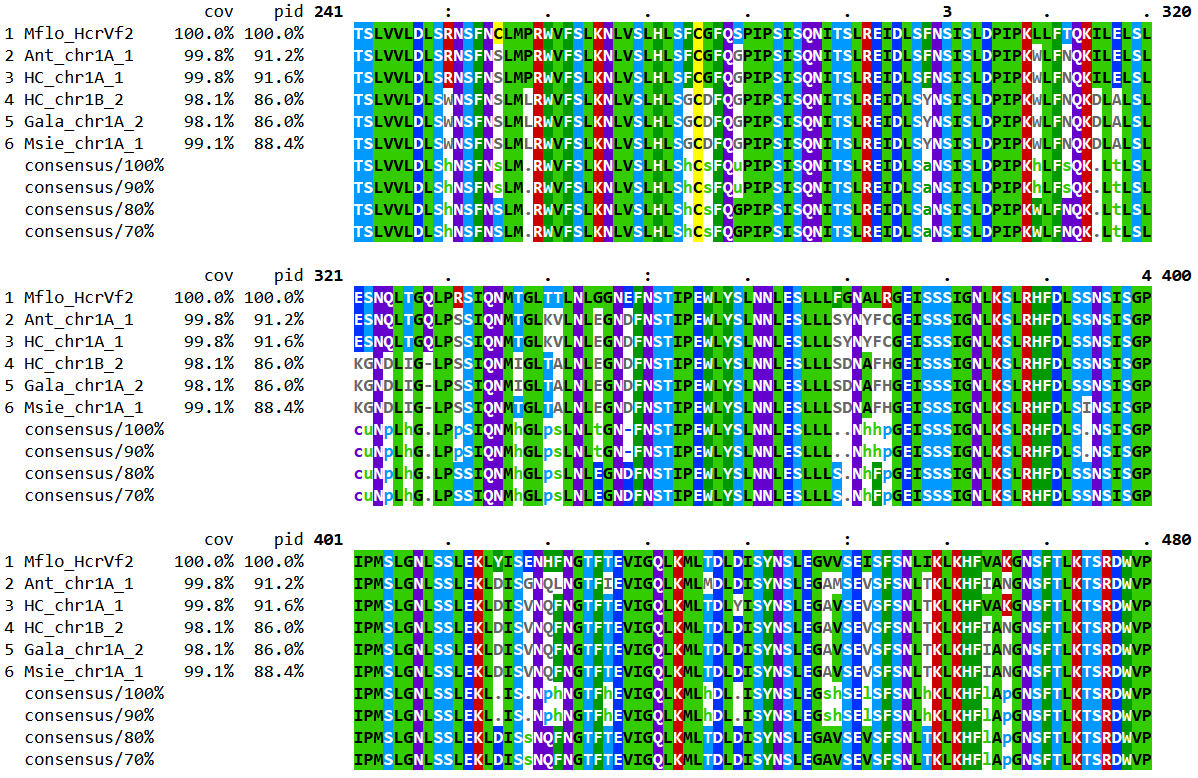
***

***
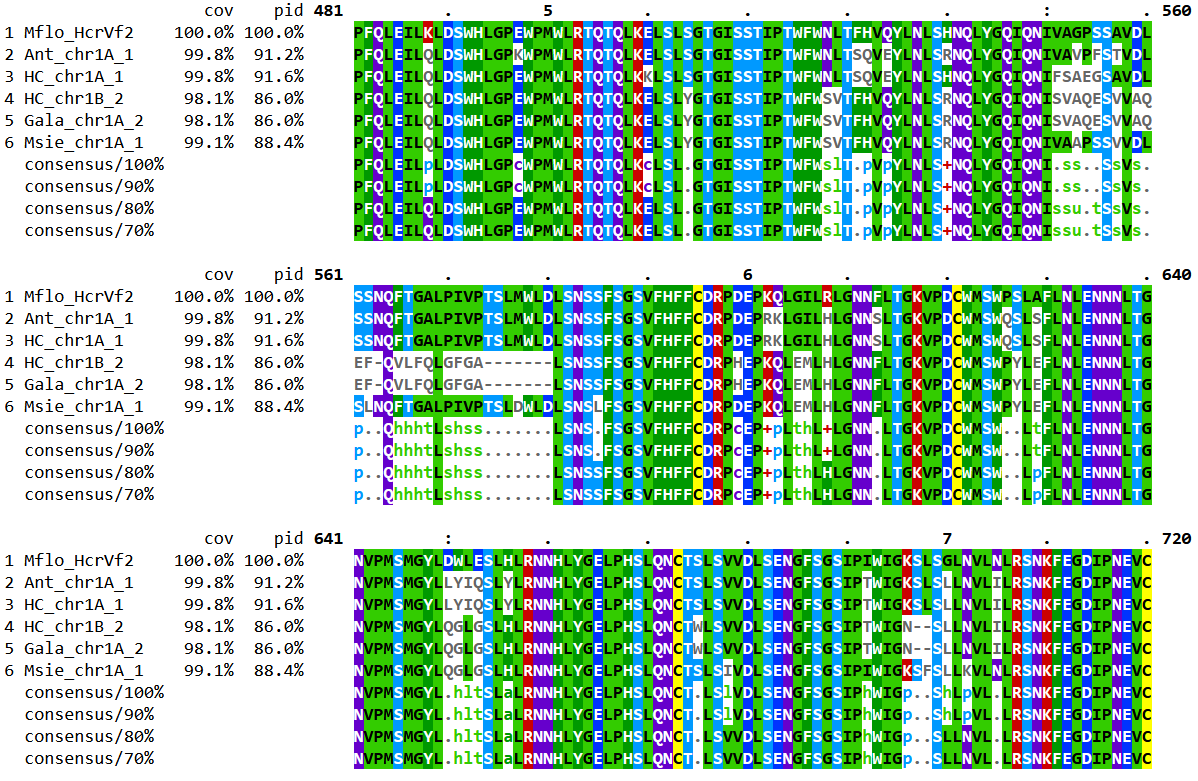
***

***
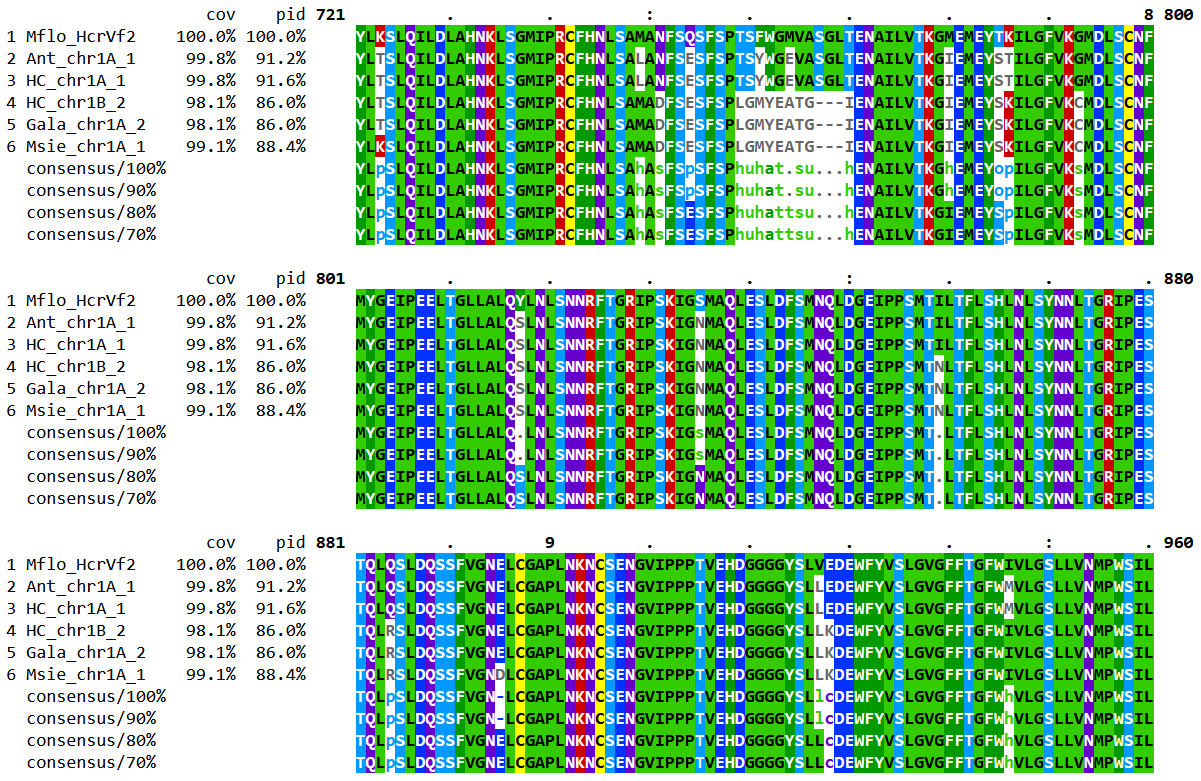
***

***
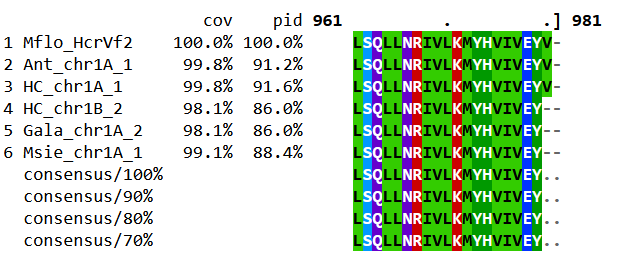
***

**Figure S13:** Alignment of the predicted amino acid sequences of HcrVf2 (Mflo_HcrVf2) and its homologs with the highest relatedness found in ‘Antonovka’ 172670-B (Ant_chr1A_1), ‘Honeycrisp’ (HC_chr1A_1 and HC_chr1B_2), ‘Gala’ (Gala_chr1A_2), and Malus sieversii (Msie_chr1A_1). Sequence identifiers indicate accession/cultivar name, Chr indicates chromosome number followed by the haplotype A and B and the subsequent number of the blast ranking. Cov: coverage; pid: identity. Identical colors indicate specific amino acid matches at specific position. “-“ indicates a missing amino acid, and “.” Indicates that no consensus can be found.

***
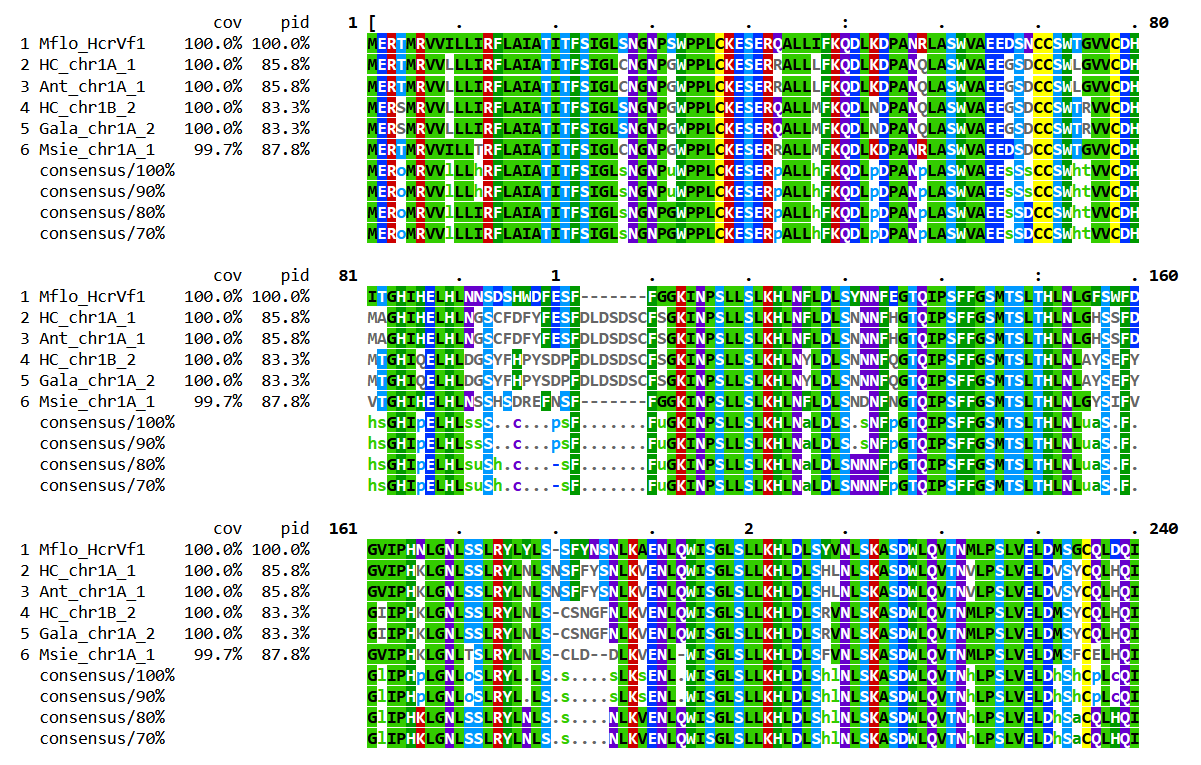
*** ***
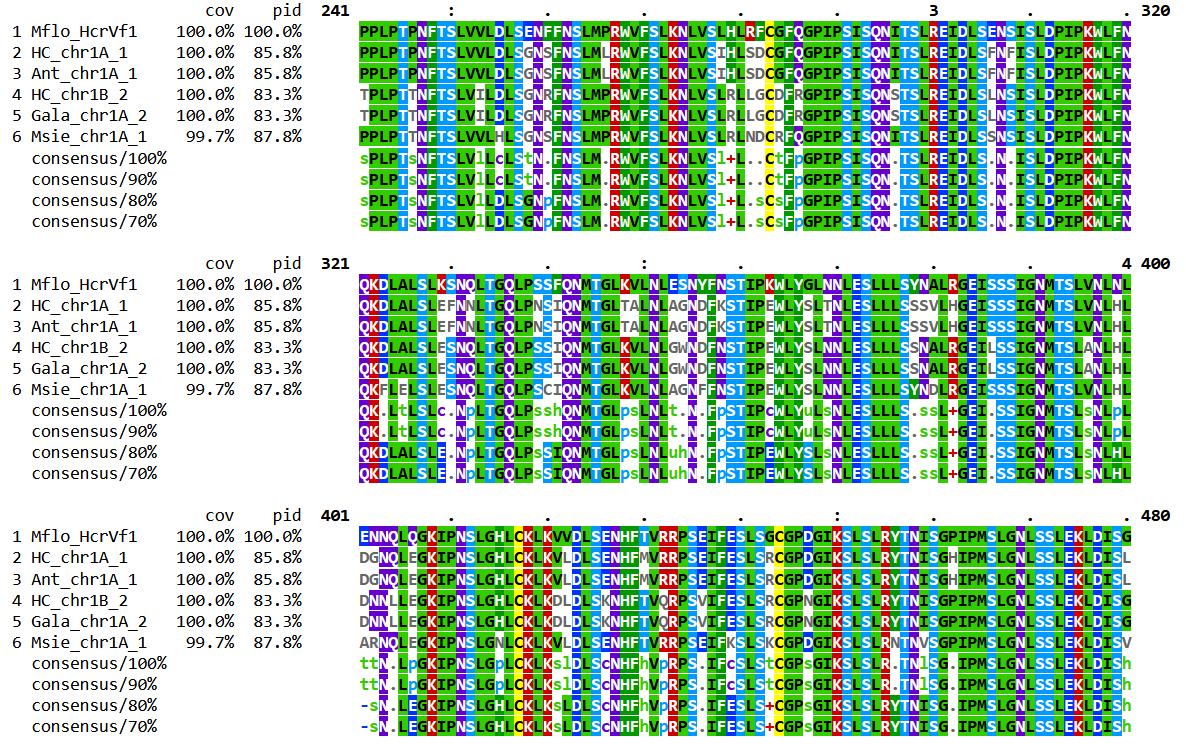
***
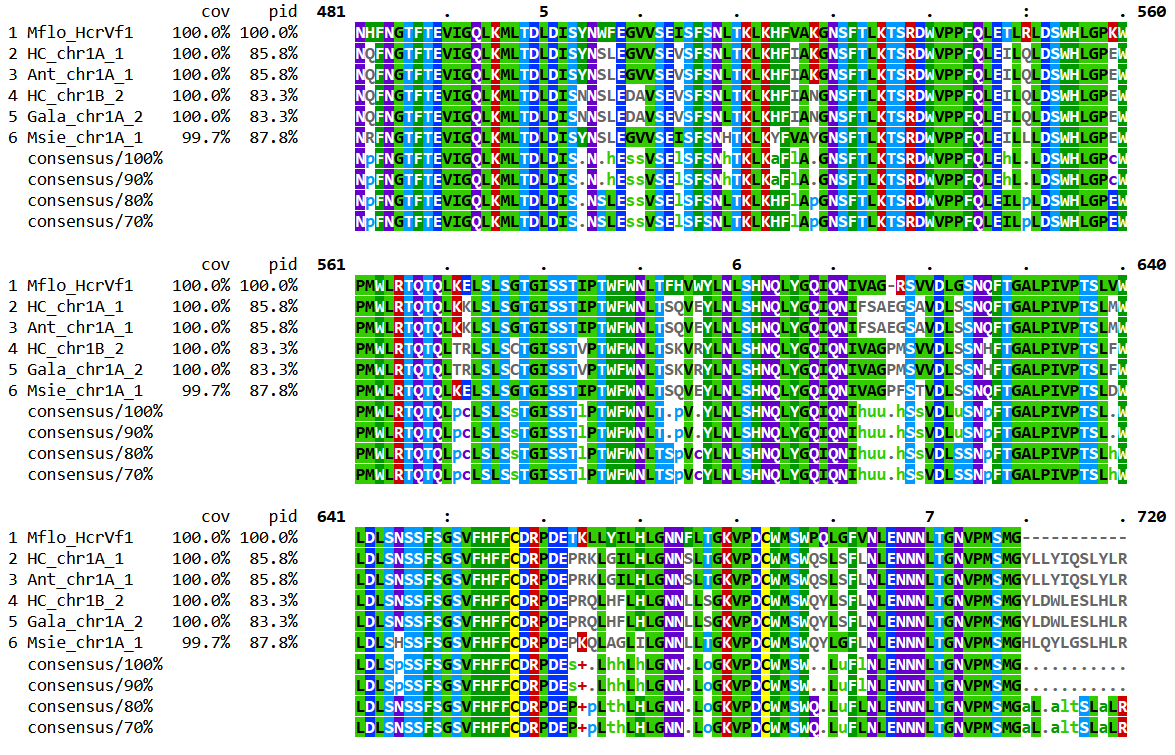

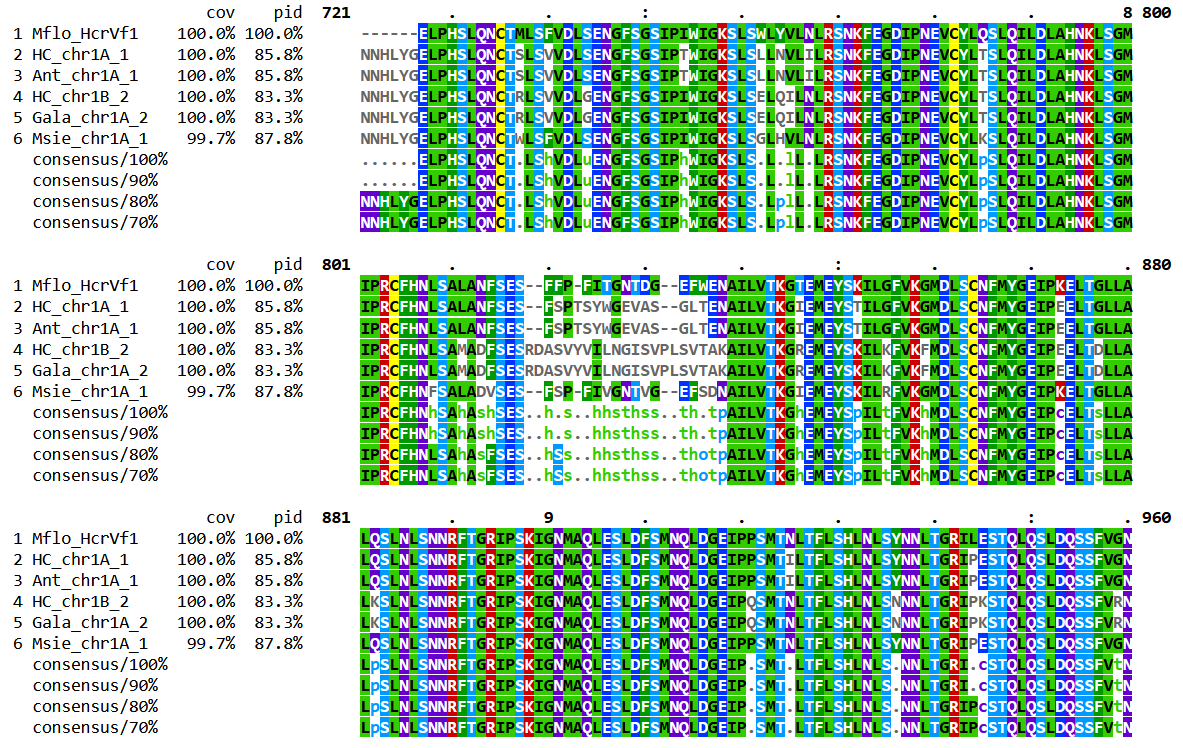

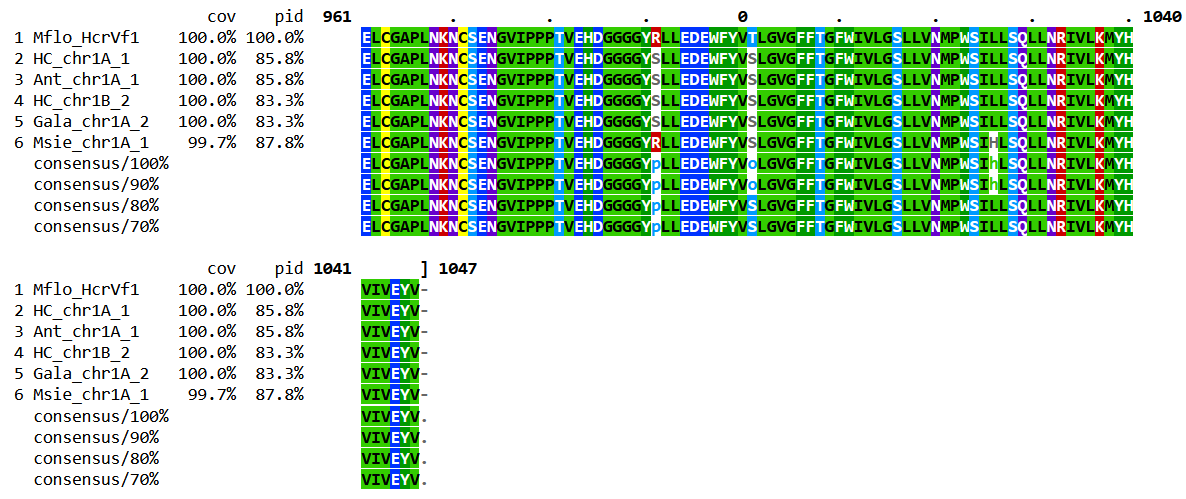


**Figure S14:** Alignment of the predicted amino acid sequences of HcrVf1 (Mflo_HcrVf1) and its homologs with the highest relatedness found in ‘Antonovka’ 172670-B (Ant_chr1A_1), ‘Honeycrisp’ (HC_chr1A_1 and HC_chr1B_2), ‘Gala’ (Gala_chr1A_2), and Malus sieversii (Msie_chr1A_1). Sequence identifiers indicate accession/cultivar name, Chr indicates chromosome number followed by the haplotype A and B and the subsequent number of the blast ranking. Cov: coverage; pid: identity. Identical colors indicate specific amino acid matches at specific position. “-“ indicates a missing amino acid, and “.” Indicates that no consensus can be found.

**Table S1:** CDS (coding sequence) of HcrVf2 from linkage group 1 of M. floribunda 821 compared with other HcrVf gene CDS. bp: base pairs

| **Name** | **GenBank accession** | **Length (bp)** | **Identity (ratio and %)** | **Coverage (%)** | **e-value** |
| --- | --- | --- | --- | --- | --- |
| HcrVf1 | AJ297739 | 3048 | 1676/1817 (92) and 1177/1268 (93) | 54 | < 10e-6 |
| HcrVf3 | AJ297741 | 2748 | 1722/1816 (95) and 945/1021 (93) | 55 | < 10e-6 |
| HcrVf4 | EU794466 | 2,889 | 1727/1816 (95) and 945/1021 (93) | 55 | < 10e-6 |

**Table S2:** The list of GRIN “PI” reference accession numbers for “Antonovka” 172670-B and apple cultivars whose SNP data from the Illumina Infinium® 20K SNP array data (Howard et al., 2021) were used to compare haplotype identities on chromosome 1 and their analyzed corresponding scab resistance gene locus.

| **Genotype** | **Analyzed resistance gene locus** | **Reference Accession ID number** | **Accession Source** |
| --- | --- | --- | --- |
| Antonovka 172670-B | *Rvi17* | PI 589956 | USDA-Geneva |
| Antonovka (OB) Obyknovennaja | *Rvi17* | / | / |
| Honeycrisp | *Vhc1* | PI 644174 | USDA-Geneva |
| Topaz | *Rvi6* | / | / |
| Liberty | *Rvi6* | PI 588943 | USDA-Geneva |
| Remo | *Rvi6* | / | / |
| Goldrush | *Rvi6* | PI 590218 | USDA-Geneva |
| Nova_Easygro | *Rvi6* | PI 588838 | USDA-Geneva |
| Golden Delicious | None | PI 590184 | USDA-Geneva |
| Gala | None | PI 392303 | USDA-Geneva |
| Idared | None | PI 588841 | USDA-Geneva |
| Fuji | None | PI 588844 | USDA-Geneva |
| McIntosh | None | PI 588817 | USDA-Geneva |

**Table S*3*:** Summary statistics for repeats of the two haplomes (Hap1: Haplome A; Hap2: Haplome B) of apple ‘Antonovka’ 172670-B.

| **Class** | | **Hap1** | | | **Hap2** | | |
| --- | --- | --- | --- | --- | --- | --- | --- |
|  |  | **Count** | **Base pairs** | **%** | **Count** | **Base pairs** | **%** |
| **Class II - DNA transposons** | **CMC-EnSpm** | 18,068 | 5,429,010 | 0.83 | 17,370 | 5,179,304 | 0.81 |
|  | **Dada** | 158 | 49,990 | 0.01 | 72 | 14,777 | 0.00 |
|  | **Ginger** | 116 | 29,237 | 0.00 | 120 | 32,312 | 0.01 |
|  | **MULE-MuDR** | 80,520 | 15,803,482 | 2.43 | 80,675 | 15,783,469 | 2.48 |
|  | **Maverick** | 111 | 28,263 | 0.00 | 98 | 16,055 | 0.00 |
|  | **PIF-Harbinger** | 45,872 | 14,051,303 | 2.16 | 45,730 | 14,085,061 | 2.21 |
|  | **TcMar-Mariner** | 147 | 36,649 | 0.01 | 81 | 18,894 | 0.00 |
|  | **TcMar-Pogo** | 1,004 | 147,317 | 0.02 | 988 | 145,683 | 0.02 |
|  | **TcMar-Stowaway** | 79 | 43,989 | 0.01 | 86 | 48,484 | 0.01 |
|  | **hAT-Ac** | 41,080 | 11,758,936 | 1.81 | 40,276 | 11,527,443 | 1.81 |
|  | **hAT-Charlie** | 1,878 | 409,890 | 0.06 | 1,909 | 408,146 | 0.06 |
|  | **hAT-Tag1** | 19,176 | 5,688,498 | 0.87 | 19,334 | 5,780,339 | 0.91 |
|  | **hAT-Tip100** | 24,332 | 5,173,279 | 0.80 | 24,381 | 5,140,977 | 0.81 |
|  | **Helitron** | 18,202 | 6,095,320 | 0.94 | 17,668 | 5,745,849 | 0.90 |
|  | **Unknown** | 52,963 | 9,162,522 | 1.41 | 53,032 | 9,161,147 | 1.44 |
| **Class I - LINE** | **CRE-Odin** | 58 | 9,251 | 0.00 | 52 | 7,942 | 0.00 |
|  | **L1** | 13,588 | 6,745,193 | 1.04 | 13,486 | 6,610,581 | 1.04 |
|  | **L1-Tx1** | 662 | 35,649 | 0.01 | 659 | 36,407 | 0.01 |
|  | **L2** | 4,139 | 754,097 | 0.12 | 4,144 | 774,375 | 0.12 |
|  | **Penelope** | 74 | 17,397 | 0.00 | 95 | 22,796 | 0.00 |
|  | **R2** | 20 | 2,091 | 0.00 | 18 | 1,952 | 0.00 |
|  | **RTE-BovB** | 12,445 | 9,118,660 | 1.40 | 12,576 | 9,270,664 | 1.46 |
|  | **Rex-Babar** | 237 | 42,781 | 0.01 | 256 | 42,351 | 0.01 |
| **Class I - LTR** | **Cassandra** | 11,195 | 2,608,764 | 0.40 | 11,278 | 2,633,268 | 0.41 |
|  | **Caulimovirus** | 2,774 | 2,925,993 | 0.45 | 2,795 | 3,038,520 | 0.48 |
|  | **Copia** | 102,322 | 83,947,038 | 12.91 | 100,332 | 82,523,787 | 12.97 |
|  | **DIRS** | 73 | 11,561 | 0.00 | 86 | 13,651 | 0.00 |
|  | **ERV1** | 203 | 18,005 | 0.00 | 207 | 18,318 | 0.00 |
|  | **ERV4** | 63 | 10,485 | 0.00 | 59 | 8,565 | 0.00 |
|  | **ERVK** | 204 | 84,836 | 0.01 | 107 | 25,885 | 0.00 |
|  | **Gypsy** | 264,084 | 178,708,886 | 27.48 | 253,352 | 170,950,621 | 26.87 |
|  | **Pao** | 485 | 204,332 | 0.03 | 510 | 224,859 | 0.04 |
|  | **Unknown** | 19,578 | 4,491,295 | 0.69 | 19,306 | 4,437,413 | 0.70 |
| **Class I - SINE** | **B2** | 382 | 24,656 | 0.00 | 365 | 23,838 | 0.00 |
|  | **L1** | 1,100 | 411,138 | 0.06 | 1,128 | 417,612 | 0.07 |
|  | **MIR** | 64 | 5,499 | 0.00 | 63 | 5,412 | 0.00 |
|  | **tRNA-RTE** | 2,967 | 304,238 | 0.05 | 2,962 | 304,113 | 0.05 |
|  | **Unknown** | 706 | 151,936 | 0.02 | 538 | 86,325 | 0.01 |
| **Satellite DNA** | | 1,035 | 189,728 | 0.03 | 1,155 | 224,666 | 0.04 |
| **Simple repeat** | | 1,904 | 358,304 | 0.06 | 1,856 | 351,871 | 0.06 |
| **Unknown** | | 217,977 | 36,111,273 | 5.55 | 215,336 | 35,692,805 | 5.61 |
| **Total repeats** | | 962,045 | 401,200,771 | 61.70 | 944,541 | 390,836,537 | 61.43 |

**Table S4:** Comparison of DNA sequences of the identified *HcrVf1* homologs across nine genome assemblies of *Malus domestica* and wild *Malus* species. The scores show the comparison of a homolog with the *HcrVf2* coding sequence, and the position indicates the location in the corresponding genome. bp: base pairs.

| **Genotype** | **Haplotype** | **Linkage group** | **Identities**  **(bp)** | **Identity**  **(%)** | **Gaps** | **Coverage**  **(%)** | **Score** | **e-**  **value** | **Start position (bp)** | **End position (bp)** |
| --- | --- | --- | --- | --- | --- | --- | --- | --- | --- | --- |
| Malus baccata | / | scaffold241 | 2868 | 92 | 63 | 94 | 4583 | 0 | 297661 | 294569 |
|  |  |  | 2831 | 91 | 105 | 93 | 4387 | 0 | 220409 | 217333 |
|  |  |  | 2839 | 91 | 84 | 93 | 4376 | 0 | 270666 | 267541 |
| Malus sieversii | A | 1 | 2867 | 92 | 60 | 94 | 4576 | 0 | 28781367 | 28784462 |
|  |  |  | 2865 | 92 | 78 | 94 | 4531 | 0 | 28843453 | 28846570 |
|  |  |  | 2842 | 91 | 100 | 93 | 4374 | 0 | 28757019 | 28760150 |
|  |  |  | 2837 | 91 | 84 | 93 | 4371 | 0 | 28655913 | 28659038 |
|  |  |  | 2790 | 89 | 80 | 92 | 4152 | 0 | 28213489 | 28210364 |
|  |  |  | 2607 | 92 | 63 | 86 | 4093 | 0 | 28593972 | 28596816 |
|  |  |  | 2727 | 87 | 101 | 89 | 3882 | 0 | 28316931 | 28319970 |
| Malus prunifolia | / | 1 | 2836 | 91 | 87 | 93 | 4447 | 0 | 25921463 | 25918395 |
|  |  |  | 2824 | 90 | 92 | 93 | 4296 | 0 | 25826079 | 25822954 |
|  |  |  | 2821 | 90 | 84 | 93 | 4295 | 0 | 25887765 | 25884640 |
|  |  |  | 2791 | 90 | 74 | 92 | 4171 | 0 | 24878926 | 24882039 |
|  |  |  | 2725 | 87 | 69 | 89 | 3890 | 0 | 24965598 | 24968714 |
| Honeycrisp | A | 1 | 2851 | 91 | 83 | 94 | 4426 | 0 | 28940293 | 28943421 |
|  |  |  | 2734 | 88 | 97 | 90 | 3982 | 0 | 28615717 | 28618779 |
| Antonovka | A | 1 | 2851 | 91 | 83 | 94 | 4426 | 0 | 28932483 | 28935611 |
|  |  |  | 2729 | 87 | 101 | 90 | 3891 | 0 | 28581132 | 28584230 |
| Malus sylvestris | B | 1 | 2840 | 91 | 78 | 93 | 4405 | 0 | 26674974 | 26678093 |
|  |  |  | 2840 | 91 | 78 | 93 | 4405 | 0 | 26681969 | 26685088 |
|  |  |  | 2792 | 89 | 80 | 92 | 4161 | 0 | 26077537 | 26074412 |
|  |  |  | 2738 | 88 | 94 | 90 | 4002 | 0 | 26207985 | 26211050 |
| Malus sylvestris | A | 1 | 2840 | 91 | 78 | 93 | 4405 | 0 | 27480509 | 27483628 |
|  |  |  | 2740 | 88 | 97 | 90 | 4005 | 0 | 26974751 | 26977819 |
| Honeycrisp | B | 1 | 2839 | 91 | 84 | 93 | 4398 | 0 | 28433144 | 28436249 |
|  |  |  | 2823 | 90 | 94 | 93 | 4283 | 0 | 28444494 | 28447631 |
|  |  |  | 2790 | 89 | 80 | 92 | 4152 | 0 | 27998850 | 27995725 |
|  |  |  | 2726 | 87 | 101 | 89 | 3877 | 0 | 28112552 | 28115650 |
| Gala | A | 1 | 2839 | 91 | 84 | 93 | 4398 | 0 | 26631036 | 26634141 |
|  |  |  | 2823 | 90 | 94 | 93 | 4283 | 0 | 26654860 | 26657997 |
|  |  |  | 2790 | 89 | 80 | 92 | 4152 | 0 | 26237152 | 26234027 |
|  |  |  | 2726 | 87 | 101 | 89 | 3877 | 0 | 26313988 | 26317086 |
|  |  |  | 2473 | 89 | 94 | 81 | 3681 | 0 | 26642394 | 26645170 |
| HFTH1 | / | 1 | 2823 | 90 | 94 | 93 | 4283 | 0 | 27964518 | 27967655 |
| Golden Delicious | / | 1 | 2823 | 90 | 94 | 93 | 4283 | 0 | 27949555 | 27952692 |
| Antonovka | B | 1 | 2823 | 90 | 94 | 93 | 4283 | 0 | 27242857 | 27245994 |
| Malus baccata | / | scaffold292 | 2790 | 89 | 74 | 92 | 4167 | 0 | 39190 | 36077 |
|  |  |  | 2738 | 88 | 90 | 90 | 4012 | 0 | 266269 | 269334 |
| Gala | B | 1 | 2790 | 89 | 80 | 92 | 4152 | 0 | 23660261 | 23657136 |
|  |  |  | 2790 | 89 | 80 | 92 | 4152 | 0 | 23708567 | 23705442 |
|  |  |  | 2725 | 87 | 101 | 89 | 3873 | 0 | 23790585 | 23793683 |
|  |  |  | 2725 | 87 | 101 | 89 | 3873 | 0 | 23796092 | 23799190 |
| Malus prunifolia | / | 17 | 2768 | 89 | 73 | 91 | 4070 | 0 | 23441270 | 23438152 |
| Malus baccata | / | scaffold580 | 2768 | 89 | 73 | 91 | 4070 | 0 | 182768 | 185886 |
